# Next Generation DNA Damage Response Inhibitors: Harnessing Nanocarriers and Tumor Microenvironment for Precision Cancer Therapy

**DOI:** 10.32604/or.2026.071632

**Published:** 2026-02-24

**Authors:** Abhishikt David Solomon, Himanshu Kumar Vats, Shivam Chowdhary, Supriya Nandlal Kanoujiya, Ajit Prakash, Hina Sultana, Sabyasachi Mohanty, Billy W. Day, Tarun Pant

**Affiliations:** 1 Division of Oral and Craniofacial Health Sciences, University of North Carolina at Chapel Hill, Chapel Hill, NC 27599, USA; 2 Department of Molecular and Cellular Engineering, Jacob Institute of Biotechnology and Bioengineering, Prayagraj, 211007, India; 3 Manipal Institute of Virology, Manipal Academy of Higher Education, Manipal, 576104, India; 4 School of Biotechnology, Jawaharlal Nehru University, New Delhi, 110067, India; 5 Department of Biochemistry and Biophysics, University of North Carolina, Chapel Hill, NC 27599, USA; 6 Integrative Program for Biological and Genome Sciences (iBGS), UNC Chapel Hill, Chapel Hill, NC 27599, USA; 7 Department of Chemical and Bio-Molecular Engineering, University of Nebraska-Lincoln, Lincoln, NE 68588, USA; 8 ReNeuroGen LLC, Milwaukee, WI 53122, USA; 9 Department of Surgery, Division of Pediatric Surgery, Medical College of Wisconsin, Milwaukee, WI 53226, USA; 10 Children’s Research Institute, Children’s Wisconsin, Milwaukee, WI 53226, USA

**Keywords:** DNA damage response (DDR), DDR inhibitors (DDRis), tumor microenvironment

## Abstract

Tumor survival, genomic stability, and therapy resistance are dictated by the DNA damage response (DDR). Although poly (ADP-ribose) polymerase (PARP) inhibitors have established the DDR as a therapeutic target, many tumors evade first-generation drugs by rewiring their adaptive repair pathways and imposing microenvironmental constraints. This review synthesizes recent discoveries in key DDR pathways, such as PARP, ataxia telangiectasia and Rad3-related kinase (ATR), ataxia telangiectasia mutated kinase (ATM), checkpoint kinase 1 (CHK1), WEE1 G2 checkpoint kinase (WEE1), and DNA-dependent protein kinase (DNA-PK), and describes the next-generation inhibitors designed to increase selectivity and circumvent resistance. We also analyze the role of hypoxia, stromal remodeling, inflammatory cytokines, and immune-cell plasticity in the tumor microenvironment in determining DDR dependency and response. Special attention is paid to cGAS-STING, immunogenic signaling via damage-associated molecular patterns (DAMPs), and mechanisms that convert a cold tumor into a hot one. Lastly, we touch upon the new nanocarrier-based delivery approaches that enhance pharmacokinetics, target resistant tumor niches, and expand the possibilities for combinatorics with immunotherapy and radiotherapy. Collectively, these findings provide a guide to the implementation of next-generation DDR inhibitors and nanomedicines to deliver a more accurate, durable, and context-specific cancer therapy.

## 1 Introduction

The DNA damage response (DDR) is a highly intricate network of cellular systems responsible for detecting, signaling, and repairing the DNA damage caused by endogenous (replication errors and oxidative stress) and exogenous factors (such as chemical, radiation, or ultraviolet (UV) light exposure) [1,2]. Disruption in DDR is a characteristic feature of cancer that contributes to genomic instability, excessive cellular proliferation, and resistance to treatment [3–5]. As a result, there has been significant research interest in developing new therapies to target weaknesses in the DDR of cancer cells.

The nucleus and mitochondria are the two primary organelles that contain DNA in mammalian cells [6]. The primary mechanisms for nucleolar DNA repair systems include direct reversal (repairing alkylation DNA, base excision repair (BER) targeting non-bulky damaged DNA bases and single strand breaks (SSBs), nucleotide excision repair (NER) for extensive helix-distorting DNA damages, insertion/deletion loop (IDL) repair and mismatch repair (MMR) for base-base mismatch repair, recombinational repair, which primarily works at DNA double strand breaks (DSBs) and is further subdivided into non-homologous end joining (NHEJ) and homologous recombination (HR) repair, alternative non-homologous end joining (alt-NHEJ), which is used to repair DSBs and translesion synthesis (TLS), which is generally a damage tolerance pathway [7].

Genomic instability is largely compromised by DSBs, which are generally the most deleterious type of DNA damage, according to mounting evidence [8]. Numerous essential DNA repair mechanisms have evolved in mammalian cells to counter various types of DNA damage throughout evolution. The NER, BER, and MMR pathways, for instance, have all been thoroughly studied [9–11]. However, aberrant DNA damage repair mechanisms and procedures have often been linked to the evolution of cancer cells. For example, Ataxia-Telangiectasia Mutated (ATM) kinases are frequently mutated in between 30% and nearly 50% of cancer cell lines, including breast, prostate, pancreatic, and lymphomas [12]. Chemotherapy resistance in cancer may be associated with these mutations [13]. Moreover, genes linked to cell cycle machinery are essential for causing cancer cells to evade the effects of chemotherapy and radiation therapy [14]. The majority of cancer cell death strategies involve either inducing abnormal HR in the G1 phase, causing mitotic arrest in cancer cells, or bypassing the cell cycle checkpoint.

The rapidly growing genomic instability of cancers also presents therapeutic opportunities to target DDR pathways, which can specifically kill cancer cells by inducing exogenous DNA damage, additional replication stress, and DDR suppression [1]. This is likely the primary reason why radiation and other DNA-damaging chemotherapeutics, such as alkylating drugs and topoisomerase inhibitors, were successful in their early stages of treatment [15]. These substances, however, tend to harm healthy tissues without discrimination, which can result in serious adverse effects [16].

Over the past few years, there has been a significant surge in publications addressing the role of genomic instability and mutations in cancer formation and pathogenesis. As a result, there have been significant preclinical and clinical investigations for developing new therapies to target weaknesses in the DDR of cancer cells. In addition, an immense body of research has focused on therapeutic opportunities to target DDR pathways, which can specifically kill cancer cells by inducing exogenous DNA damage, additional replication stress, and DDR suppression [1]. For instance, the ATM inhibitor (ATMi) KU-55933 has been widely used *in vitro* and *in vivo* investigations to advance the preclinical and clinical use of ATMs [17]. Also, ATR inhibitors (ATRis) like AZ20, VE-821, and VE-822 have demonstrated high selectivity over several other DDR kinases [18]. WEE1 inhibitor (WEE1i), adavosertib (MK1775), has also shown promising potential in combination therapies in clinical use [19,20]. However, their activity is limited to several resistance mechanisms that enable cancer cells to survive and proliferate, thereby activating compensatory signaling pathways. The progress and clinical development of ATMis KU-55933, CP466722, KU-60019, and KU-59403 demonstrated minimal cytotoxicity but exhibited off-target effects and poor pharmacokinetics when used alone. They faced obstacles due to their context-dependent efficacy, which is influenced by factors such as p53 status and mode of delivery [21]. Consequently, CHK1 inhibitors (CHK1i) such as UCN-01 and DNA-PK inhibitors (DNA-PKis) like wortmannin and KU-0060648 face specificity challenges and unfavorable pharmacokinetics (PK), poor stability, and low solubility [22,23].

This review examines the recent progress in targets and inhibitors within the DDR space. We discuss the development of specific inhibitors of DDR proteins by leveraging various modes of delivery, including nanotechnology and antibody-drug conjugates (ADCs), to enhance drug delivery and effectiveness [24,25]. Additionally, we discuss the challenges of conventional DDR inhibitors and the potential use of next-generation DDR inhibitors as a therapeutic strategy to combat genomic instability, which may prevent cancer growth and pathogenesis.

## 2 Overview of DNA Damage Response Initiation, Repair, and Replication Mechanisms

**Figure 1:**
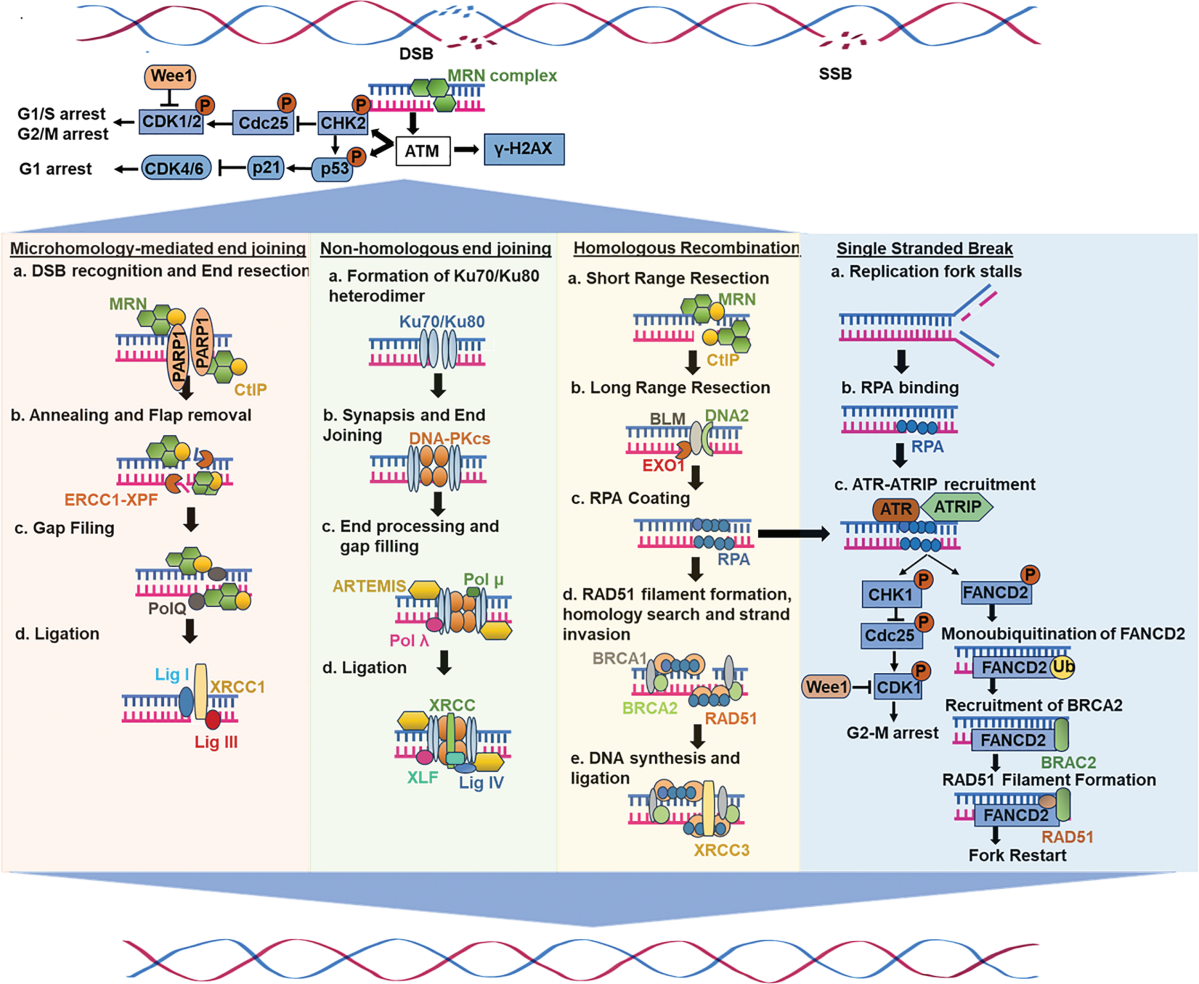
A general scheme of DNA damage response and repair mechanisms

Several studies have substantiated that the genomic instability is comprised mainly of DSBs, which are generally the most deleterious type of DNA damage. Numerous essential DNA repair mechanisms have evolved in mammalian cells to counter various types of DNA damage throughout evolution. This section explicitly discusses the current understanding of the mechanisms associated with DNA damage. Fig. 1 illustrates the mechanisms of DNA damage from the preclinical and clinical studies.

### 2.1 ATM Kinases in DSB Repair

Ataxia telangiectasia (A-T) is a rare inherited autosomal recessive condition characterized by immune system deficiency, hypersensitivity to radiation, increased cancer susceptibility, and progressive neurodegeneration, leading to impaired movement coordination (ataxia) [26–28]. A-T arises from ATM gene mutations that disrupt ATM kinase function, a pivotal regulator of the DDR [29]. Structurally akin to phosphoinositide 3-kinase (PI3K), ATM belongs to the phosphatidylinositol 3-kinase-related kinase (PIKK) family [28,30,31].

As a serine/threonine kinase, ATM acts as a master coordinator in DDR, governing DBS repair through HR, NHEJ, and cell cycle checkpoints [32,33]. Upon DNA damage, ATM undergoes self-activation (autophosphorylation) at serine 1981, relocating to damaged sites [34]. Thereafter, it phosphorylates crucial targets like checkpoint kinases (e.g., CHK2), the histone variant H2AX, and the mediator of DNA damage checkpoint 1 (MDC1) [34–36], triggering cell cycle arrest, activation of DNA repair pathways, or programmed cell death (apoptosis) [37]. ATM-mediated regulation of the G1/S, S, and G2/M checkpoints involves pathways such as the ATM-p53-p21, ATM-Chk2-CDc25, and ATM-BRCA1-Cyclin B1 pathways [38,39] (Fig. 1).

ATM influences both NHEJ and HR, but studies suggest a more prominent role in NHEJ, where it phosphorylates the DNA-dependent protein kinase catalytic subunit (DNA-PKcs), thereby impacting the accuracy and efficiency of DSB repair [40–42]. ATM also interacts intricately with A-T and Rad3-related (ATR), which predominantly responds to replication stress and SSBs [43,44]. Since ATR inhibition prompts ATM compensation, a dual-targeting approach involving ATM and ATR inhibitors (ATMis and ATRis) may offer a more promising therapeutic strategy for cancer treatment [36,45]. The WEE1 inhibitor (WEE1i) adavosertib (MK1775) has also shown promising potential in clinical use [19,20].

Early ATMs faced significant challenges, including feeble blood-brain barrier (BBB) penetration, which limited their use in brain tumors, such as glioblastomas (GBMs) [46]. Many compounds also lacked specificity, leading to off-target toxicity and a narrow therapeutic window. KU60019 exhibited potential to radiosensitize glioma-initiating cells (GICs), which provided the first evidence supporting ATM inhibition as a cancer treatment [47,48]. ATM’s restricted success highlighted its therapeutic potential, prompting the development of brain-penetrant inhibitors with improved sensitivity and safety profiles. The insights from these lessons have shaped the development of next-generation ATMs, which exhibit improved pharmacokinetics and demonstrate clinical potential.

### 2.2 ATR-CHK1 Axis in DNA Damage Response and Replication Repair

ATR, a key DDR kinase, responds to replication stress and SSBs by activating checkpoint kinase 1 (CHK1), leading to cell cycle arrest and DNA repair [49]. ATR inactivation increases replication stress, leading to DSB formation and cancer cell death [36]. ATRis are explored as monotherapies or in combination with immune checkpoint inhibitors (ICI), Poly(ADP-ribose) polymerase (PARP) inhibitors (PARPis), and DNA-damaging agents (DDAs) [50,51]. By disrupting the ATR/CHK1 pathway, ATRis promote genomic instability and mitotic catastrophe [52]. ATR inhibition also sensitizes cancer cells to replication stress-inducing agents by preventing ssDNA from causing cell cycle arrest [53].

CHK2, a serine/threonine kinase, regulates the cell cycle and DDR, particularly in response to replication stress caused by chemotherapy or repair deficiencies [54,55]. CHK1 inhibition sensitizes cancer cells to genotoxic treatments, particularly in those with G1-S checkpoint defects that depend on intra-S and G2/M checkpoints for survival [56]. Oncogene amplification on extrachromosomal DNA (ecDNA) is associated with a poor prognosis and resistance to therapy. These circular DNA elements exhibit high genomic plasticity but also induce elevated replication stress, making ecDNA-amplified tumors highly dependent on CHK1 [54]. Consequently, the application of CHK1 inhibitors is a promising strategy against such malignancies [57].

### 2.3 Non-Homologous End Joining (NHEJ)

NHEJ involves several mechanistically distinct stages in the repair of DSBs [28]. Upon DSB formation, the Ku70/80 heterodimer binds DNA ends, initiating the assembly of the NHEJ machinery [58,59]. Ku serves as a recruitment hub for downstream NHEJ components [28] (Fig. 1). Furthermore, DNA-PKcs, a major protein kinase, forms the DNA-PK holoenzyme upon binding to DNA-bound Ku [60–62]. DNA binding activates this process, which results in the phosphorylation of NHEJ components [63]. DNA-PKcs autophosphorylation represents an essential phase in NHEJ-mediated DSB repair processes, while its functional consequences await clarification. Moreover, for ligation to occur, DNA ends in the synaptic complex must be closely aligned, facilitated by NHEJ factors like X-ray repair cross-complementing protein 4 (XRCC4), XRCC4-like factor (XLF), and PAXX [64–66]. Finally, DNA ligase IV catalyzes ligation, forming a complex with XRCC4, and can tolerate terminal mismatches and damaged bases [67]. However, many DNA end structures cannot be directly ligated. As a result, many end-processing factors, such as nucleases and polymerases, are attracted to DSBs and work on the ends to prepare them for ligation. Deficits in this pathway have several detrimental effects on human health, as NHEJ is essential for repairing both spontaneous and planned DSBs [28]. Defective V(D)J recombination causes severe combined immunodeficiency (SCID) when mutations occur in the genes encoding XLF, LIG4, DNA-PKcs, and Artemis [28]. Human cells with abnormalities in essential NHEJ components are typically hypersensitive to infrared radiation, and a fraction of SCID patients also show radiosensitivity [28].

### 2.4 Homologous Recombination (HR)

Homologous recombination (HR) ensures genome stability by enabling error-free repair of DSBs, interstrand crosslinks (ICLs), and DNA gaps before and after DNA replication [68]. HR, which is mainly active in the S and G2 phases [69], heavily relies on homologous strands, typically the sister chromatid, as repair templates in somatic cells [70]. The process involves two key steps: DNA strand invasion and the homology search, which ensures precise repair. HR initiation requires extensive 5^′^ to 3^′^ end resection, generating 3^′^-OH single-stranded DNA (ssDNA) tails, facilitated by the MRN (MRE11-RAD50-NBS1) complex that also triggers DDR [71] (Fig. 1). C-terminal-binding protein-interacting protein (CtIP), an interacting partner of MRN, leads to its activation, shifting its role from DNA damage sensing to resection [72,73]. Exonuclease 1 (EXO1), endonuclease DNA2, and Bloom syndrome helicase further extend long-range resection [74–76]. Following resection, replication protein A (RPA) coats ssDNA, preventing secondary structures and nuclease degradation. RPA also inhibits RAD51 filament formation and nucleation, thereby preventing its interaction with ssDNA ends [77]. Thereafter, several mediators, including PALB2, RAD51 paralogs, and BRCA2, evict RPA through interactions with BRCA1 and BRCA1-associated RING domain protein 1 (BARD1), promoting RAD51 nucleofilament formation [77–79]. RAD51 recombinases search for a homologous sequence to facilitate strand invasion, forming a D-loop (displacement loop) intermediate. This structure stabilizes invading strands, allowing DNA polymerases-mediated synthesis using the homologous template. Following strand extension, the D-loop is resolved through one of the two pathways: synthesis-dependent strand annealing (SDSA), resulting in non-crossover-repair, or the double Holliday junction (dHJ) pathway that would lead to crossover or non-crossover outcomes [80,81]. Overall, these mechanisms collectively ensure high-fidelity DNA repair and error-free bypass, preventing mutations and maintaining genome stability [82].

## 3 Recent Advances and Challenges in Targeting DNA Damage Response Using Small Molecule Inhibitors

**Figure 2:**
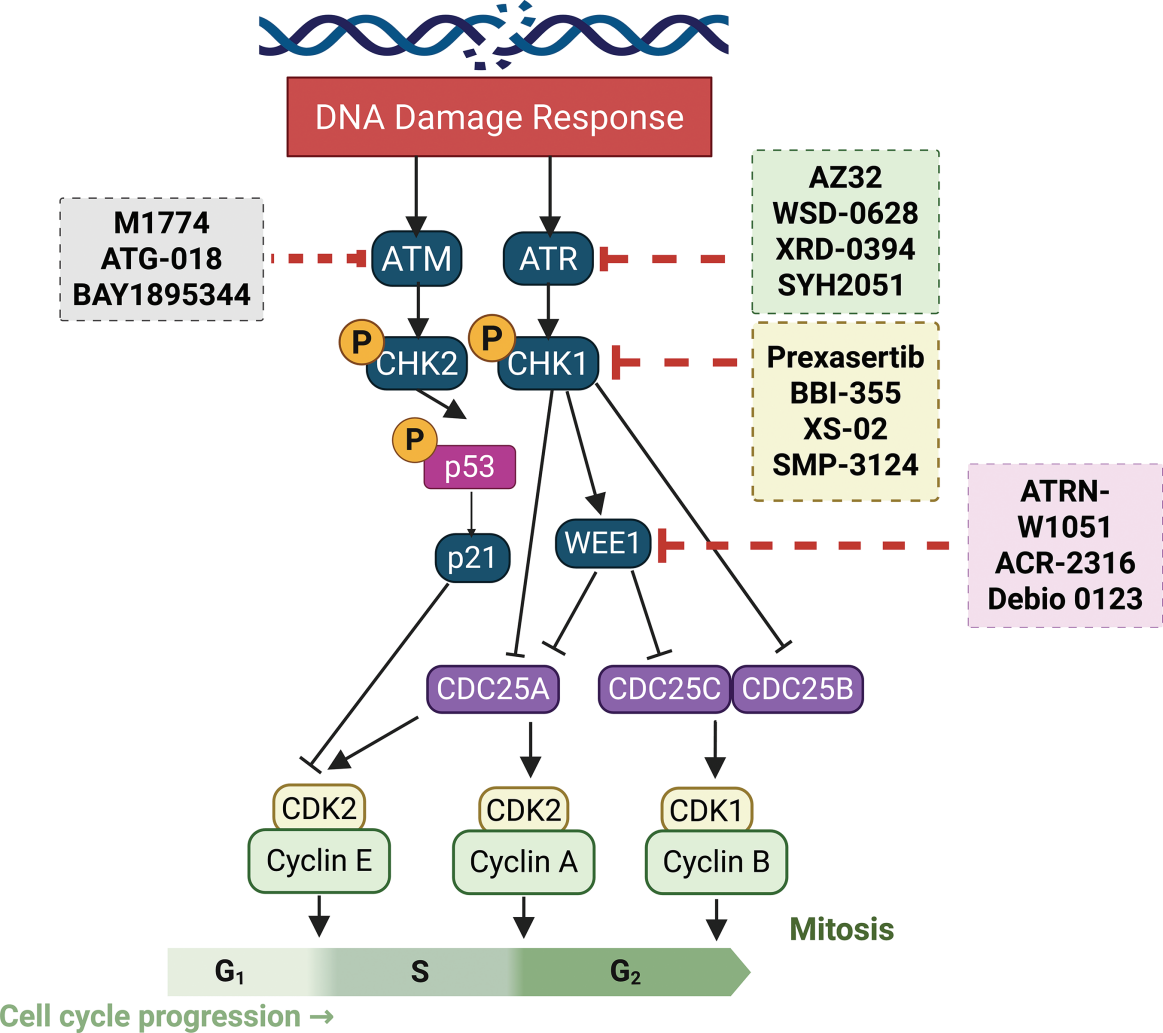
Illustration of current clinical and pre-clinical drug models targeting DNA damage response signaling

The above-described studies indicate that genomic instability is mainly comprised of DSBs, which are the most deleterious type of DNA damage. Conventional cancer therapies like radiation and chemotherapy exert their cytotoxic effects by directly damaging DNA [83,84]. However, cancer cells frequently harbor robust DNA repair mechanisms enabling them to survive and proliferate despite such damage [85,86]. This inherent resistance has fueled the development of small-molecule inhibitors (SMIs) targeting key DDR proteins [87]. Therefore, DNA damage inhibitors have been intensively investigated using animal models and cell culture-based studies (Fig. 2). Several classes of DDRis are currently under investigation, each with distinct mechanisms of action and therapeutic applications. The current section will examine preclinical and clinical drug models targeting DDR signaling using distinct DNA damage inhibitors like ATM, ATR, CHK1/2, Wee, and DNA-PK. Different preclinical and clinical studies investigating DDRi are summarized in (Table 1).

**Table 1:** Different preclinical and clinical studies investigating DDRi

DDR inhibitors	Inhibitor types	Experimental models	Dosage	Mechanism of action	Pharmacological properties and pharmacokinetics	Clinical stage	Limitations
ATM inhibitors	CP466722	1. *In vitro* (human fibroblasts, cancer cell lines). 2. Short-term cell culture.	2–8 μM for ATM inhibition (IC_50_ ≈ 370 nM); rapid onset, reversible effect	1. Selective inhibitor of ATM kinase. 2. Blocks ATM-dependent phosphorylation and cell cycle checkpoint signaling, radiosensitizes cells, reversible and non-toxic.	Effective *in vitro* inhibition for upto 8 h of treatment before recovery	*In vitro*	1. Limited potency and selectivity. ATM activity recovered after removal of ATM inhibitor 2. Only *in vitro* application 3. Not suitable for *in vivo* studies due to low metabolic stability
	WSD-0628	1. *In vitro*: MCF7, U251 glioblastoma cell lines. 2. *In vivo*: (i) mouse xenograft models (brain tumors, melanoma) (ii) oral and intravenous administration.	1. Oral dosing range: 0.1 to 10 mg/kg (mice) 2. IV dosing 1 to 10 mg/kg; plasma half-life ~18 h	1. Potent, selective ATM kinase inhibitor (IC50 ~0.1 nM); crosses blood-brain barrier. 2. Radiosensitizer via inhibition of ATM-mediated DDR modest inhibition of DNA-PK and ATR at higher concentrations	1. Orally bioavailable and CNS (brain) penetrant in preclinical rodent models; unbound brain-to-plasma partition coefficient (Kp, uu) ~0.3 in mouse and ~0.44 in rat. Displays greater-than-dose-proportional exposure in plasma and brain (i.e., exposure increases more than dose), which suggests non-linear clearance. 2. Minimal efflux transporter (P-gp/BCRP) liability at the blood-brain barrier; brain tissue distribution uniform across regions.	Phase 0/1a	1. Limited to human PK and preclinical studies only. 2. Non-linear pharmacokinetics will also affect dose scaling, making extrapolation to human trials more difficult. 3. Because of its mechanism of action and ability to penetrate the brain, Fyn hydro phosphorylation would be expected to enhance DNA-damage signaling in normal tissues (e.g., CNS), especially during radiotherapy.
	AZ32	1. *In vitro* (human glioma, colon carcinoma lines with p53 mutant and wildtype). 2. *In vivo* (orthotopic mouse glioblastoma models, oral gavage).	1. Oral: 100–200 mg/kg in mice. 2. Plasma/brain concentrations exceed ATM cell IC_50_ (~0.3 μM) for 22 h after single dose.	Selective ATM kinase inhibitor penetrates blood-brain barrier, blocks ATM-dependent DNA damage response, radiosensitizes p53-mutant tumor cells, reduces phosphorylation of ATM substrates (p53, KAP-1) after irradiation.	-	-	-
	ZN-B-2262	1. Preclinical (SW60 colorectal cancer cell line, *in vitro* and *in vivo* xenograft mouse models) 2. Administered with irinotecan and radiotherapy	Dosage details limited; reported as effective in combination with irinotecan and radiation in tumor growth inhibition.	Selective ATM kinase inhibitor impairs tumor DNA damage repair by inhibiting ATM signaling, enhances chemo- and radiosensitivity.		Early-phase clinical studies (for advanced solid tumors, including glioblastoma)	1. The selectivity and toxicity challenges. 2. Preclinical to clinical translation is impeded by the limited pharmacokinetics/ADME data, making it difficult to ascertain how well the tumor exposures correlate with *in vivo* human settings.
ATM/DNA-Pk inhibitors	XRD-0394	1. *In vitro*: (human and murine cancer cell lines). 2. *In vivo*: (mouse xenograft, orthotopic models); oral administration in preclinical and phase I clinical studies.	1. *In vitro*: Active at nanomolar concentrations 2. *In vivo* (mouse): effective oral doses 12–50 mg/kg daily	1. Potent, orally bioavailable dual ATM and DNA-PKcs inhibitor 2. Abrogates DNA damage response; radiosensitizes tumors, enhances PARPi and topoisomerase I inhibitor effects, shows single-agent activity (esp. in BRCA mutant models)	1. Orally-bioavailable. 2. in human Phase I (single dose 160 mg) the median t_max_ was about 2.3 h and mean terminal t½ ~11.1 h. 3. Plasma concentrations remained above the preclinical target (~530 ng/mL ≈1 µM) for ~15 h.	Completed a first-in-human Phase Ia/1 trial (NCT05002140) in combination with palliative radiotherapy in advanced cancer patients.	1. Single dose/tolerability only. 2. Full multi-dose safety, with normal tissue radio sensitization risk. 3. Theoretical risk of enhanced toxicity in normal tissues when combined with radiotherapy due to dual inhibition of ATM and DNA-Pk.
ATR inhibitors	ATG-018	1. Preclinical *in vitro* (multiple human solid tumor and hematologic cancer cell lines). 2. *in vivo* mouse xenograft models; oral administration in early clinical trials.	No specific dose mentioned	1. Selective and potent ATR kinase inhibitor. 2. Blocks ATR-dependent DNA damage response signaling, leading to synthetic lethality especially in tumors with replication stress and DDR defects.		Entered Phase I (first patient dosed in August 2022) in an open-label, dose-finding study of monotherapy in advanced solid tumors and hematologic malignancies (trial NCT05338346)	1. Early stage so far (monotherapy dose-escalation only. 2. Biomarker-driven patient selection is still immature. 3. Lack of public PK/PD exposure-response data, and there is potential for normal-tissue toxicity given ATR’s role in replication stress and normal cell cycles.
	SMP-3124	1. Evaluated in multiple preclinical xenograft models (subcutaneous, peritoneal dissemination, and orthotopic ovarian cancer models, including ES-2 and patient-derived xenografts). 2. Administered as a liposomal formulation for targeted delivery and prolonged retention.	Numerical doses not stated	1. Selective CHK1 inhibitor that induces S-phase arrest, replication stress, and apoptosis in tumor cells. 2. Liposomal encapsulation enhances tumor targeting and sustained pharmacodynamic CHK1 inhibition.	1. Liposomal nanomedicine with durable pharmacodynamic response, longer plasma retention, and higher tumor accumulation. 2. Well-tolerated, no significant weight loss or systemic toxicity observed in preclinical models.	1. Phase 1/2 clinical study ongoing in adults with advanced solid tumors. 2. Investigational New Drug (IND) filed/planned for 2024.	1. Limited to preclinical and early-phase clinical data; long-term safety and PK in humans not yet reported 2. Potential liposome-related delivery variability and an unknown human toxicity profile require further validation.
	M1774	1. Nanomolar doses (superior efficacy over ceralasertib and berzosertib). 2. Oral administration. 3. Synergistic dosing with TOP1/2 inhibitors, cisplatin, and talazoparib under evaluation.	1. Nanomolar doses (superior efficacy over ceralasertib and berzosertib). 2. Oral administration; synergistic dosing with TOP1/2 inhibitors, cisplatin, and talazoparib under evaluation.	1. Potent oral ATR inhibitor; blocks ATR/CK1 checkpoints, enhancing TOP1 inhibitor–mediated tumor cell death by preventing replication arrest and inducing DNA damage. 2. Proteomic profiling shows upregulation of G2/M and replication-associated proteins (PLK1, CCNB1, TIPIN, CDC45, TIMELESS, RPA1). Demonstrates synergy with DNA-damaging agents and overcomes chemoresistance in SLFN11-deficient cells.	Oral administration; median t_max_ ≈ 0.5–3.5 h and mean terminal t½ ≈ 1.2–5.6 h (exposure-related target engagement ≥130 mg QD).	Phase I dose-escalation (RDE 180 mg QD, 2 weeks on/1 week off)	2. Limited single-agent objective responses (activity enriched in specific DDR-mutant tumors). 3. Need to define optimal combination schedules and longer-term safety.
WEE1 inhibitors	Adavosertib (MK1775)	*In vitro* (cancer cell lines) and *in vivo* (mouse xenograft), Phase I/II/III trials in humans as monotherapy and combo (with radiation, gemcitabine, carboplatin, paclitaxel, etc.) 2. 150–200 mg/m^2^ in combination with chemo/radiotherapy. 3. Single oral doses up to 300 mg in PK studies.	1. Monotherapy MTD typically 225 mg BID for 2.5 days every 21 days (humans).	Selective inhibitor of Wee1 kinase. Disrupts G2/M and intra-S checkpoint control, increases CDK1 (CDC2) and CDK2 activity, leads to premature mitosis and cell death in replicating cancer cells (especially with DDR defects or p53 mutations), and sensitizes to DNA-damaging agents.	Median time to C_max_ ~2.2–4.1 h; mean half-life ~5–12 h following oral dosing.	Phase I and II trials	1. Dose-limiting toxicities: myelosuppression, diarrhea, fatigue, nausea. 2. The efficacy of the narrow therapeutic window is schedule-dependent when co-administered with chemotherapy. 3. The response is variable across different tumor types and identifying effective biomarkers (e.g., TP53 mutation) for patient selection remains challenging.
	ATRN-W1051	1. *In vitro* (ovarian cancer cell lines) 2. *In vivo* (xenograft models of Cyclin E1-amplified high-grade serous ovarian cancer, CCNE1-HGSOC)	1. Inhibits WEE1 with an IC_50_ = 2.2 nM. 2. suppresses ovarian cancer cell proliferation at 100–200 nM.	1. Selective WEE1 inhibitor designed to minimize off-target toxicity. 2. Does not inhibit PLK1-3; reduces growth of CCNE1-amplified HGSOC tumors in xenograft models with favorable pharmacokinetics and reduced systemic toxicity.	Reportedly favorable PK in preclinical species achieves similar AUC_0–24_ to AZD1775/ZN-c3 at 3–8× lower dose, and is orally bioavailable with tolerable daily dosing in mice	1. IND-enabling/pre- IND stage per company disclosures. 2. Preclinical development with plans for future clinical evaluation.	1. Evidence only in animals no human PK/safety data yet; translation risk that mouse PK/tox will not predict human exposure or tolerability. 2. Class-wide hematologic toxicities remain a concern despite improved selectivity and must be confirmed in humans. 3. Requires 3–8× lower doses than AZD1775 or ZN-c3 for comparable exposure; orally administered, well-tolerated with daily dosing.
	ACR-2316	1. *In vitro* (19-cell-line proliferation screen) 2. *Ex vivo* (12 ovarian cancer PDX models) 3. *In vivo* (xenograft mouse models)	1. Potent WEE1 inhibition (IC_50_ = 2 nM; IC_90_ = 10 nM). 2. Moderate PKMYT1 inhibition (IC_20_ = 44 nM). 3. Orally administered with dose-dependent tumor inhibition and good tolerability.	1. Dual WEE1/PKMYT1 inhibitor optimized via co-Crystallography; selectively activates mitotic kinases (CK1, CDK2, PLK2/3); induces S-G2/M accumulation. 2. Demonstrates superior activity and selectivity over adavosertib and lunresertib; robust tumor growth inhibition in xenograft models.	1. Orally bioavailable with a half-life suitable for once-daily dosing in preclinical species. 2. Plasma exposures in rats/dogs and mice are consistent with the projected human exposure required for activity.	IND-enabling stage/ preclinical	1. Potential hematologic toxicities remain a translational concern despite an encouraging preclinical hematologic profile. 2. Residual translational uncertainty: superior selectivity and preclinical efficacy do not guarantee clinical benefit, or tolerability requires human PK/PD, dose scheduling, and biomarker validation.
	Debio 0123	1. *In vitro* (primary GBM cell lines).2. *In vivo* (mice, rats, and monkeys); orthotopic and subcutaneous GBM xenograft models. 3. Oral, once-daily administration in patients (21-day cycles, continuous dosing). 4. Preclinical activity in multiple *in vitro* cancer cell lines and *in vivo* human xenograft models; brain-penetrant.	1. Efficient brain penetration with brain-to-plasma ratios: 0.6 (mice), 1.52 (rats), 4 (monkeys).2. Tumor-to-plasma ≈ 0.62; combination with TMZ induced complete regressions in 75% of mice for 100 days. 3. Oral bioavailability pH dependent. 4. Dose escalation from 30 to 350 mg once daily in Phase-1; MTD = 260 mg QD; target engagement reached from ≈200 mg; steady state reached 15–21 days.	1. Selective WEE1 inhibitor with strong CNS penetration. 2. Enhances TMZ- and radiation-induced DNA damage and apoptosis; suppresses GBM growth up to 73.7% (brain) and 57.5% (subcutaneous). 3. Orally active and well tolerated; food has minimal effect on absorption, but PPIs should be avoided. 4. Potent, highly selective WEE1 kinase inhibition → abrogates S/G2 and G2/M checkpoints (CDC2/CDK1 deregulation) → mitotic catastrophe and tumor cell death.	1. Oral, brain-penetrant, linear PK with dose-proportional plasma exposure between 150–350 mg; steady-state after ~15–21 days. 2. Continuous exposure required for optimal monotherapy efficacy. 3. On-target pharmacodynamic engagement demonstrated by reduced pCDC2 in skin biopsies (≥200 mg).	1. First-in-human Phase 1 (NCT05109975) dose-escalation completed to RP2D 2. Dose-expansion planned/ongoing in biomarker-selected cohorts (accrual in US and Switzerland).	1. Early data: small cohort, short median treatment (median 6 weeks). 2. Need for longer follow-up to define efficacy, safety profile (cardiac QT risk), and biomarker predictiveness.
CHK1 inhibitors	Prexasertib	*In vitro* (human tumor cell lines, including ovarian and squamous cell carcinoma); *in vivo* (mouse xenograft models); clinical trials (patients with relapsed/refractory cancers).	Preclinical IC50: 2–10 nM; Clinical dosing: 105 mg/m^2^ IV every 2 weeks.	Potent and selective CHK1 inhibitor; blocks CHK1 kinase activity, abrogates cell cycle checkpoints, induces replication stress, DNA damage, and apoptosis, especially in p53-deficient tumors.	1. multicompartment, dose- and time-independent PK with no meaningful inter-cycle accumulation. 2. systemic exposures at the recommended adult dose (105 mg/m^2^ given as a 1-h IV infusion every 14 days) reach the predicted therapeutic range from preclinical models.	Completed first-in-human dose-escalation/ expansion (RP2D 105 mg/m^2^ q14d) and progressed into Phase-2 studies (including NCI-sponsored ovarian cancer trials and multiple disease-specific phase-2 efforts).	1. Frequent, predictable hematologic toxicity (transient Grade-4 neutropenia common. 2. Febrile neutropenia reported), modest monotherapy activity limited to subsets (e.g., some platinum-resistant ovarian patients), and need for validated predictive biomarkers and optimized combination/timing to balance efficacy vs. normal-tissue toxicity.
	XS-02	1. Evaluated *in vitro* in multiple cancer cell lines (e.g., OVCAR3) and *in vivo* using OVCAR3 and MDA-MB-436 xenograft and patient-derived xenograft (PDX) mouse models. 2. Oral administration used for *in vivo* studies.	1.IC_50_ = 2 nM (CHK1) and 282 nM (CHK2) in enzyme assays. 2. *In vivo* showed dose-dependent tumor inhibition (exact mg/kg not stated) with no significant body weight loss.	1. Selective CHK1 inhibitor with moderate CHK2 inhibition. 2. Blocks CHK1/CHK2 phosphorylation, impairs DNA damage checkpoint activation, enhances replication stress, and synergizes with PARP inhibitor (olaparib) to promote tumor regression.	1. Orally bioavailable, well-tolerated, and showed broad antitumor efficacy across xenografts. 2. Acceptable oral bioavailability across multiple non-clinical species. 3. Good tolerability and pharmacodynamic target engagement were demonstrated.	1. Preclinical candidate. 2. Investigational New Drug (IND) application planned for 2023 (not yet in human trials at the time of report).	1. No human PK or toxicity data yet; preclinical only. 2. Requires validation of efficacy and safety in clinical settings. 3. Potential on-target toxicities (CHK1 inhibition–related hematologic or replication stress effects) remain to be evaluated.
	BBI-355	1. *In vitro* (ecDNA-positive tumor cell lines); *in vivo* (mouse xenograft models). 2. Phase I/II POTENTIATE clinical trial evaluating monotherapy and combinations with erlotinib (EGFR inhibitor) and futibatinib (FGFR inhibitor) in solid tumors with oncogene amplifications.	1. Dose-escalation cohorts used Q2D (every-other-day oral) dosing with planned/observed dose levels including 20 mg, 40 mg, 60, 80 mg Q2D and intermittent schedules tested (examples: 120 mg 2-on/5-off; 80 mg 2-on/5-off; 80 mg 3-on/11-off) in the POTENTIATE protocol.	1. Selective CHK1 inhibitor targeting replication stress in ecDNA-positive cancers (harboring MYC, EGFR, FGFR2 amplifications). 2. Inhibits CHK1 to disrupt replication-stress response, inducing tumor cell death. Enhances tumor suppression when combined with FGFR or CDK4/6 inhibitors. pCHK1-S345 validated as a pharmacodynamic biomarker confirming target engagement in preclinical and clinical settings. 3. Prevents ecDNA-mediated resistance to targeted therapies.	1. Pharmacology: oral, small-molecule CHK1 inhibitor with demonstrated on-target PD activity (pCHK1-S345 induction) in preclinical tumors and in skin and tumor biopsies from patients on POTENTIATE. 2. Orally bioavailable. 3. Dose-dependent increase in plasma exposure reported in clinical PK analyses presented in company/abstract material. 4. No peer-reviewed paper with full numeric human PK parameters (t½, CL, AUC) is available in the cited abstracts.	1. First-in-human Phase 1/2 (POTENTIATE; NCT05827614) dose escalation (single agent) and combination expansions (e.g., with EGFR or FGFR inhibitors for EGC). 2. Active/enrolling with initial pharmacodynamic proof-of-mechanism reported.	Hematologic adverse events (expected on-target class effects) have been observed in trials/abstracts necessitating careful scheduling and combination design.
DNA-Pk inhibitors	Ku-DBis (Ku-DNA binding inhibitors)	1. *In vitro* (NSCLC cell lines treated with IR, bleomycin, etoposide) 2. *In vivo* (NSCLC xenograft models)	Nanomolar potency; optimized oxindole derivatives within X80 core improved uptake and selectivity.	1. Inhibits Ku70/80–DNA binding, blocking DNA-PK recruitment and NHEJ initiation. 2. Reduces DNA-PKcs autophosphorylation, activates the ATM–p53 signaling pathway, and sensitizes tumor cells to DSB-inducing agents. 3. Enhances DDR suppression and decreases non-specific protein interactions.	1. Improved cellular uptake and reduced protein binding in optimized compounds. 2. First demonstration of *in vivo* DNA-PK inhibition (autophosphorylation blocked, DDR modulated).	Preclinical; first *in vivo* efficacy shown in xenograft models.	1. Single-agent activity variable; BRCA1-deficient cells resistant or antagonistic with DSB therapies 2. Clinical translation untested; human PK, safety, tolerability, and efficacy remain unknown.
	SY-7021	1. *In vitro* (MDA-MB-468 breast cancer, NHEJ reporter assay). 2. *In vivo* (NCI-H1703 xenografts, oral dosing)	Oral administration twice daily; 60 mg/kg achieved 105.6% TGI; high selectivity (~400× over ATM/ATR).	1. Selective DNA-PKi that dose-dependently suppresses NHEJ efficiency, enhances γH2AX, CHK2, and p53 phosphorylation, and induces G2/M arrest and apoptosis. 2. Potent antitumor activity alone or with DOX; favorable PK and safety.	1. Good oral bioavailability 2. Dose-dependent tumor inhibition *in vivo*.	Preclinical tested *in vitro* and *in vivo* xenograft models.	1. Clinical safety and efficacy not yet evaluated. 2. Monotherapy may be less effective in some tumor types. 3. Combinatorial therapy with DNA-damaging may be required for maximal effect.
	Peposertib (M3814)	1. *In vitro* (TNBC and cervical cancer cell lines) 2. *In vivo* (xenograft models, IR, and chemo combinations)	1. In the xenograft cervical cancer model (HeLa flank in mice): 50 mg/kg orally, once daily (5 days/week, weeks 1–3) in combination with 2 Gy ×5 radiation per week. 2. In the TNBC xenograft study: tested dosing of peposertib 50 mg/kg QD, 100 mg/kg QD, and 100 mg/kg BID, orally, for 4 days/week in combination with pegylated liposomal doxorubicin (PLD). 3. In a Phase Ib combination chemoradiotherapy study (rectal cancer): peposertib doses tested were 50 mg, 100 mg, 150 mg, and 250 mg once daily (QD) in combination with capecitabine and radiotherapy.	1. Orally available DNA-PK inhibitor that blocks catalytic activity and enhances tumor sensitivity to DSB-inducing treatments. 2. In combination with IR or chemotherapy, induces tumor regression and pro-inflammatory immune responses. 3. Supports use in radiotherapy.	1. Orally bioavailable, well-tolerated in mice. 2. Enhances radiation or chemotherapy efficacy without severe toxicity. 3. Demonstrated tumor regression and radio sensitization in xenograft models.	1. Preclinical 2. Supporting further clinical evaluation in combination with DNA-damaging agents (TNBC, cervical cancer, HNSCC models).	1. Efficacy depends on p53 and HPV tumor status; some tumors (p53-wt/HPV-) show relapse after combination therapy. 2. Clinical safety, tolerability, and dosing in humans not yet established.
		KU-0060648	1. *In vitro* (human breast cancer MCF7, colon cancer SW620 cells). 2. *In vivo* (mouse xenograft models MCF7, SW620), intravenous and oral administration.	1. IC_50_ for DNA-PK inhibition: ~0.02 μM (MCF7) and ~0.2 μM (SW620). 2. 12.5 mg/kg i.v. in mice xenograft studies. 3. 1 μM in cell culture for growth inhibition and chemo sensitization.	1. Dual inhibitor of DNA-PK and PI3K with higher specificity for DNA-PK 2. Inhibits DNA-PK auto-phosphorylation, suppresses DNA damage repair, enhances cytotoxicity of chemo drugs etoposide and doxorubicin, delays tumor growth	1. After a 10 mg/kg dose in mice (various routes: i.v., i.p., p.o.), bioavailability via p.o. was reported as ≥100% (compared to i.v.) and via i.p. was ~78%. 2. The compound also achieved tumor concentrations in a mouse xenograft model that maintained the level of *in vitro* activity (i.e., >~1 µM) for ≥~4 h post-dose.	1. The efficacy is cell line-dependent (differential DNA-PK vs PI3K activity) 2. weak PI3K inhibition in some cell lines (SW620). 3. Due to dual DNA-PK/PI3K activity, predictive biomarkers (such as PIK3CA status) are likely needed to guide patient selection. 4. The translational challenges broadly observed for DDR inhibitors (potency, selectivity, toxicity and optimal scheduling with chemotherapy/radiotherapy).
	BAY-8400	1. *In vitro* (DDR-deficient cancer cells). 2. *In vivo* (prostate cancer xenografts. 3.PSMA–thorium-227 TAT models).	1. In the *in vivo* prostate tumor-bearing mice study (with PSMA-targeted thorium-227 conjugate), administered orally at doses up to 175 mg/kg daily, with no body-weight loss or overt toxicity in the tested animals. 2. 150 mg/kg p.o. achieved increased antitumor efficacy in combination models.	1. Triazoloquinoxaline-derived DNA-PKi, optimized for DNA-PK selectivity over PI3K. 2. Inhibits DSB repair, causing genomic instability and apoptosis. 3. Synergizes with targeted α-therapy (TAT), enhancing cytotoxicity in NHEJ-reliant tumors. 4. Improves therapeutic window.	1. Highly selective 2. Oral bioavailability demonstrated in rats/mice (~22% in rats.) 3. Rat IV clearance ~2.2 L/h/kg, t½ ~0.84 h	Preclinical only (*in vivo* efficacy in xenograft models, no human trials yet reported).	1. Monotherapy activity is weak (IC_50_ ~2540 nM in HT-29 cells), efficacy is mainly when combined with DNA-damage therapies. 2. Translation to human safety, tolerability, and optimal combination strategy is not yet established.
	WNC0901	1. *In vitro* (U251 glioma, A549 lung, HT29 colon cells). 2. *In vivo* (preclinical radiation-sensitization models)	*In vitro* IC50: 32.7 nM for DNA-PKcs autophosphorylation inhibition; maximal radiosensitization at 300 nM. *In vivo*: 25–50 mg/kg orally for effective radiosensitization; higher doses (50 mg/kg) doubled tumor control duration.	1. Selective inhibition of DNA-PK catalytic subunit (DNA-PKcs). 2. Blocks NHEJ-mediated DNA repair, enhancing cytotoxicity of radiation-induced DNA double-strand breaks.	1. Wistar rat (IV 2 mg/kg, PO 20 mg/kg): Oral bioavailability 116%, t½ 2.6 h, clearance 33 mL/min/kg, moderate Vss 1.32 L/kg, low brain penetration (Kpuu 2.6%). 2. Beagle dog (IV 2 mg/kg, PO 20 mg/kg): Oral bioavailability 131%, t½ 4.28 h, moderate clearance 8.5 mL/min/kg, Vss 1.87 L/kg, fraction unbound 74.3%. Stable in liver cytosol (t½ >2000 min), low aldehyde oxidase liability.	1. Preclinical only 2. Evaluated in cell lines and mouse xenograft/PDX models.	1. No human safety or tolerability data yet. 2. Monotherapy activity is modest. 3. Efficacy relies on a combination with radiation. 4. Potential radiosensitization of normal tissues (oral mucositis and weight loss observed at higher doses in mice).

### 3.1 ATM Inhibitors

Several small-molecule ATMis are in development or undergoing clinical trials, exhibiting varying degrees of potency and selectivity [33,88]. Many feature bioisosteric variants or imidazoquinolinone cores to enhance efficacy and reduce off-target effects [89]. Combination strategies are being explored, including WSD-0628 with radiation for glioblastoma (GBM) and melanoma brain metastases [90] and ZN-B-2262 with topoisomerase inhibitor-based ADCs [91]. Since ATM repairs topoisomerase-induced DNA breaks, its inhibition may enhance cancer cell death. Additionally, AZD1390, a novel ATM kinase inhibitor, simultaneously targets HR and microhomology-mediated end joining (MMEJ), increasing TMZ cytotoxicity and radiosensitivity in resistant GBMs [92]. Further validation is needed to optimize ATM inhibition and overcome treatment resistance. Therefore, there is a need to validate several aspects of ATM inhibition, as well as multiple targets that may completely abrogate cancer cell proliferation and treatment resistance.

#### 3.1.1 WSD-0628

WSD-0628, designed by Wayshine Biopharmaceuticals and evaluated by the Mayo Clinic, is an effective ATMi reported to date, effectively inhibiting ATM activity in U251 glioblastoma cells at a concentration of 30 nM. In combination with IR, it showed highly significant survival rates (%) in GBM xenograft mice and demonstrated strong brain penetration [90,93]. Investigations by Rathi et al. reported a potent inhibition of ATM autophosphorylation and downstream targets CHK2 and KAP1 at 100 nM and reduced γH2AX foci in GBM43 cells. Additionally, preclinical studies using intracranial p53-mutant GBM43 xenograft models demonstrated rapid CNS distribution and prolonged retention at a dose of 10 mg/kg, compared to lower doses [94]. Oral dosing revealed non-linear pharmacokinetics, and only minor increases in brain exposure were observed in efflux transporter KO mice, suggesting limited BBB resistance [94]. It is potent, and its CNS accessibility supports its ongoing clinical evaluation as a powerful radiosensitizer for recurrent, advanced-grade gliomas (NCT05917145).

#### 3.1.2 AZ32

AZ32 is an orally bioavailable ATM inhibitor that blocks DDR and radiosensitizes gliomas. A study by Karlin et al. demonstrated the efficacy of AZ32 as a potential penetrant of the BBB [46]. It enhanced radiosensitivity in intracranial gliomas, outperforming AZ31 in syngeneic orthotopic glioma models. Its efficacy was particularly notable in glioma cell lines harboring mutant p53 or checkpoint defects, where combining AZ32 with low-dose radiation induced over a sixfold increase in tumor apoptosis compared to healthy brain tissue [46]. Furthermore, McCabe et al. revealed that ATM inhibition is synthetically lethal in PTEN-deficient tumors, which exhibit chronic ATM activation, elevated DNA damage, and heightened reactive oxygen species (ROS) levels. ATM blockade in these cells triggered apoptosis, irreversible DNA damage, and cell cycle arrest, whereas wild-type cells remained less affected [95]. These findings highlight AZ32 inhibitor as a promising therapeutic agent, particularly in tumors with p53 mutations or PTEN loss, warranting further clinical evaluation.

#### 3.1.3 XRD-0394

XRD-0394 functions as a sophisticated and potent dual inhibitor targeting ATM and DNA-PKcs, demonstrating increased tumor cell death when combined with IR, topoisomerase inhibitors, and PARPis, especially in BRCA1/2-deficient cells. This compound demonstrates independent-agent performance while exhibiting advantageous pharmacological properties. Gilmer et al. reported the completed Phase 1a trial and the identification and preliminary characterization of XRD-0394 [96]. The clinical potential of NCT05002140 emerges in combination therapies, warranting further investigation. XRD-0394 emerged as a solution to RT limitations by dual-inhibition of ATM and DNA-PK, thereby enhancing tumor sensitivity while countering resistance pathways. Preclinical data demonstrated that transient dual inhibition enhances tumor cell death while sparing non-cancerous cells in the absence of radiation. In a phase 1a trial, XRD-0394 was well tolerated with no dose-limiting toxicities when combined with palliative RT. At 160 mg, plasma levels exceeded preclinical efficacy thresholds for over 15 h, with confirmed ATM inhibition in tumor tissues [96]. Its pharmacokinetic profile supports co-administration with RT, providing a strong rationale for future trials combining XRD-0394 with RT, PARPis, immunotherapy, or topoisomerase-targeting ADCs.

#### 3.1.4 SYH2051

Clinical investigations of the novel ATMi SYH2051 are underway. ZhongQi Pharmaceutical Technology Co. developed SYH2051 [21]. Following the successful completion of pre-clinical assessment, SYH2051 has entered clinical trials both as monotherapy and in combination therapy with IR [97]. The ongoing trial, NCT06011291, aims to characterize the pharmacokinetic profile and assess therapeutic efficacy in solid tumors, particularly in head and neck cancers. With the ability to enhance the effectiveness of current DNA-damaging treatments and circumvent resistance mechanisms, ATMis constitute an exciting intervention in cancer therapies. The role of ATMs in the treatment of cancer and other illnesses will likely be further defined by ongoing research and clinical trials, despite the obstacles that remain. Personalized cancer treatment and better patient outcomes are made possible by the ability to target the DDR precisely.

### 3.2 ATR Inhibitors

The ATR/CHK1 pathway is crucial for DDR and disrupted by ATRis, leading to the accumulation of DSBs, particularly in tumor cells with high replication [52,98]. This disruption induces mitotic catastrophe, genomic instability, and cell death. ATRis also prevent ssDNA from triggering cell cycle arrest, thereby enhancing cancer cell sensitivity to replication-stress-inducing agents. Several ATRis are currently in clinical trials, both as monotherapies and in combination with chemotherapeutics, aiming to identify predictive biomarkers for personalized treatment and evaluate their therapeutic efficacy [49,53].

#### 3.2.1 M1774

M1774, an oral ATR inhibitor in clinical development, exhibits superior efficacy over ceralasertib and berzosertib in small cell lung cancer (SCLC), inhibiting cancer cell survival at nanomolar doses [52]. By blocking ATR/CK1 checkpoints, M1774 enhances TOP1 inhibitor-mediated tumor cell death by avoiding replication arrest and inducing DNA damage. Proteomic analysis revealed M1774, and SN-38 upregulate G2/M-related and replication proteins (PLK1 and CCNB1, TIPIN, CDC45, TIMELESS, and RPA1). It also synergizes with TOP1/2 inhibitors, cisplatin, and talazoparib, overcoming chemoresistance in SLFN11-deficient cells, positioning SLFN11 as a potential biomarker, and demonstrating therapeutic synergy with DNA-damaging agents in SCLC and colon cancer organoids, as well as in H82 SCLC xenografts. While promising, further evaluation of dosing and combination strategies is essential [52].

#### 3.2.2 ATG-018

The antitumor effects of ATG-018 have also been demonstrated *in vitro* and *in vivo* [98]. In a study by Hui et al., normal peripheral blood mononuclear cells viability was unaffected by ATG-018; however, in 137 out of 143 cell lines, including solid tumors and hematologic malignancies, its IC_50_ ranged from 0.22 to 10 µM [98]. Numerous genetic alterations that can be used as predictive markers have been linked to ATG-018 sensitivity. Inhibiting downstream CHK1 phosphorylation in HT-29 cells required an IC50 of 1.4 nM, whereas ATR kinase activity required 16 nM [98]. Additionally, in CDX mouse models of OCI-LY-19 (lymphoma), OE21 (esophageal cancer), and LoVo (colorectal cancer), ATG-018 demonstrated dose-dependent anticancer effects [99].

#### 3.2.3 BAY1895344

BAY1895344 is another ATRi that has shown promise in preclinical and clinical studies. It was developed by optimizing a quinoline-based scaffold with initial weak ATR inhibition [100]. Chemical refinements enhanced its potency, selectivity, and oral bioavailability, establishing it as a strong clinical candidate. Preclinical studies also showed its efficacy both as monotherapy and in combination with DDAs, particularly in tumors with DNA repair deficiencies [100]. When combined with topoisomerase inhibitors (topotecan, irinotecan), it has been shown to exacerbate DNA damage and impair DNA repair, leading to enhanced tumor cell death [101]. Also, pairing with emcitabine or cisplatin showed greater cytotoxicity by blocking DNA repair of lesions induced by these agents [102]. Moreover, with PARPi talazoparib, it has been shown to disrupt DNA repair in HR-deficient tumors [103,104].

Another investigation by Tang et al. demonstrated that BAY1895344 represses ATR-CHK1 signaling, activates the Cyclin-dependent kinase-Speckle-type POZ (CDK1-SPOP) axis, and destabilizes programmed death-ligand 1 (PD-L1) protein in prostate cancer cells [105]. BAY1895344, combined with anti-PD-L1 therapy, robustly activated innate immunity while producing a synergistic T-cell-dependent therapeutic response in syngeneic mouse models [105]. The study outcomes justify the integration of ATR-targeted therapies with ICIs for treating castration-resistant prostate cancer patients. BAY1895344 is currently undergoing clinical evaluation in patients with advanced solid tumors and lymphomas (NCT03188965). This phase 1/2 trial is assessing safety, PK, and efficacy, both alone and in combination. Biomarker analysis within trials aims to refine patient selection and optimize therapeutic outcomes [106].

### 3.3 CHK1 Inhibitors

CHK1 inhibitors (CHK1i) function by blocking cell cycle checkpoints, specifically the intra-S phase and G2/M checkpoints [107]. By inhibiting CHK1, these inhibitors allow cells to bypass the checkpoint and proceed through the cell cycle despite damaged DNA, thereby preventing arrest in response to replication stress or DNA damage [108]. In the end, this results in cell death, genomic instability, and mitotic catastrophe [102]. CHK1 makes tumor cells more vulnerable to radiation and chemotherapy, among other DNA-damaging substances [109]. This is because cancer cells are more dependent on CHK1 for survival, as they frequently have defects in multiple DNA repair pathways [110]. Specific genetically altered cancer cells may be synthetically deadly when CHK1 is inhibited. Cancer cells that harbor ATM mutations, for instance, would be especially vulnerable to CHK1 inhibition [111]. Numerous clinical trials have assessed CHK1 as a standalone treatment and in conjunction with other cancer treatments [112,113]. Although the preclinical activity of specific CHK1 inhibitors has been encouraging, their clinical development has proven difficult [114].

#### 3.3.1 Prexasertib

Prexasertib, a dual CHK1/CHK2 inhibitor, has shown clinical efficacy in platinum-resistant, BRCA wild-type high-grade serous ovarian carcinoma [115]. However, its intravenous delivery poses toxicity and compliance challenges [116]. Resistance mechanisms include IGF-1/insulin pathway activation, immunosuppressive responses, a prolonged G2 delay via reduced CDK1/Cyclin B1 activity, and shielding cells from mitotic catastrophe [115]. Despite resistance, CHK1’s role in RAD51-mediated HR remains unchanged, allowing prexasertib to sensitize resistant cells to DNA-damaging agents like gemcitabine and hydroxyurea (HU). In triple-negative breast cancer (TNBC), epidermal growth factor receptor (EGFR) signaling contributes to resistance by phosphorylating BAD, inhibiting apoptosis. EGFR blockade restores BAD activity, enhancing prexasertib sensitivity [117].

#### 3.3.2 BBI-355

BBI-355 exploits the vulnerability of cancers harboring ecDNA that encodes oncogenes such as MYC, EGFR, and fibroblast growth factor receptor 2 (FGFR2) [118,119]. ecDNA amplifications drive RS and resistance to targeted therapies through rapid, non-Mendelian oncogene distribution [54,57]. By inhibiting CHK1, BBI-355 disrupts the RS response and induces tumor cell death [120]. It has shown strong preclinical efficacy *in vitro* and *in vivo*. It selectively inhibits ecDNA-positive tumor cells and induces RS [54,118]. Oral administration in mice demonstrated dose-dependent target engagement, as evidenced by increased pCHK1-S345 levels, and robust tumor regression in ecDNA-positive models [118]. Combination therapy with FGFR or CDK4/6 inhibitors further enhances tumor suppression [118,119].

Furthermore, pCHK1-S345 has emerged as a reliable pharmacodynamic biomarker for CHK1 inhibition by BBI-355 [57]. It has been validated *in vitro*, in xenografts, and in patient skin biopsies from the ongoing POTENTIATE trial, confirming clinical target engagement and helping define optimal dosing. The phase 1/2 POTENTIATE trial evaluates BBI-355 alone or in combination with erlotinib (EGFR inhibitor) and futibatinib (FGFR inhibitor) in patients with solid tumors harboring oncogene amplifications. Initial results show good tolerability [57,118,119]. Initial results show good tolerability, manageable hematologic adverse events, dose-dependent exposure, and on-target pCHK1 modulation [119]. Since ecDNA-mediated resistance to targeted therapies poses a significant clinical challenge, BBI-355 prevents such resistance when combined with EGFR or FGFR inhibitors in preclinical gastric cancer models, yielding durable responses [118,119].

#### 3.3.3 XS-02

XS-02 is a new, orally bioavailable inhibitor of CHK1 with modest activity against CHK2. It exhibits strong anticancer activity across a variety of solid cancers, including those resistant to PARPis, with manageable side effects [116]. By inhibiting both CHK1 and CHK2 kinases *in vitro*, XS-02 reduces the phosphorylation of CHK1 entirely and CHK2 partly in OVCAR3 cancer cells. Dose-dependent suppression of tumor development is induced by oral administration of XS-02 alone [116]. In an animal model of acquired resistance to olaparib developed from patient-derived tumor xenografts, the synergy between XS-02 and olaparib, a PARPi, has been demonstrated to expedite tumor regression [116].

#### 3.3.4 SMP-3124

SMP-3124 is an innovative, liposome-encapsulated, and specific CHK1i [55]. Drug distribution is altered by this liposomal formulation, which results in extended drug retention in plasma and accumulation in tumors [55]. SMP-3124 demonstrates a strong anticancer property in several subcutaneously xenografted animals without any adverse effects [55]. In orthotopic and peritoneal dissemination models utilizing human ovarian cancer cells, it also increases overall survival [55]. Gemcitabine and SMP-3124 work together to synergistically reduce tumor development without causing any extra hematological effects [55].

Although several CHK1 inhibitors are currently under development, issues with toxicity, patient stratification, and resistance mechanisms remain. For CHK1i to realize its most significant therapeutic potential, future studies should focus on identifying predictive biomarkers, developing innovative combination strategies, and improving drug-delivery techniques. BBI-355, a new ecDNA-directed treatment, shows promise for targeting CHK1 in malignancies driven by extrachromosomal DNA oncogene amplification [118]. CHK1i’s safety and effectiveness in treating different types of cancer will be further clarified by ongoing clinical trials, such as the POTENTIATE study by Hansen et al. [118].

### 3.4 WEE1 Inhibitors

WEE1 regulates the G2/M transition by phosphorylating CDK1 at tyrosine 15 (Y15), preventing cell division in cells with damaged DNA [121]. When DNA damage occurs, WEE1 is activated to maintain the G2/M checkpoint, and its inhibition increases CDK1 activity, causing cells to bypass DNA repair, enter mitosis prematurely, and undergo mitotic catastrophe and cell death [122]. This makes WEE1 a key target in cancer treatment, especially in p53-deficient cancers, which are highly reliant on the G2/M checkpoint [123]. WEE1 inhibition induces apoptosis and mitotic catastrophe in these cells [124].

However; WEE1 inhibitors have previously been associated with severe hematological consequences; such as anemia; neutropenia; and thrombocytopenia; limiting their effectiveness [125]. Current strategies focus on developing more potent and selective inhibitors with improved pharmacokinetics to reduce off-target effects and extend the treatment window; thereby improving patient outcomes [122].

#### 3.4.1 ATRN-W1051

ATRN-W1051 is a selective WEE1i developed to reduce toxicity [125]. According to reports by Vacca et al., it has shown potential to inhibit WEE1 with an IC_50_ of 2.2 nM and suppress ovarian cancer cells at 100–200 nM concentrations [125]. Unlike AZD1775, it does not inhibit PLK-1-3, minimizing off-target effects. ATRN-W1051 also offers favorable pharmacokinetics, requiring 3–8 times lower doses than AZD1775 to achieve similar exposure in ZN-c3. It effectively reduces tumor growth in Cyclin E1-amplified high-grade serous ovarian cancer (CCNE1-HGSOC) xenograft models. It is well tolerated with daily oral dosing, highlighting its potential as a target therapeutic for CCNE1-overexpressing HGSOC.

#### 3.4.2 ACR-2316

ACR-2316 is a dual WEE1 and PKMYT1 inhibitor optimized via co-crystallography for improved single-agent activity and selectivity. In a study by Wigerup et al., ACR-2316 demonstrated potent inhibition of WEE1 at an IC_50_ of 2 nM and IC_90_ of 10 nM, following the moderate inhibition of PKYMT at IC_20_ of 44 nM, when compared to adavosertib and lunresertib, which resulted in superior activation of mitotic kinases CK1, CDK2, and PLK2 [126]. Selectivity profiling through AP3 (200+ kinases) and KINOMEscan (468 kinases) confirmed its improved specificity over existing WEE1/PKYMT1 inhibitors. Also, cell cycle analysis revealed greater S-G2/M accumulation than with adavosertib or lunresertib. Moreover, ACR-2316 showed the highest potential in a 19-cell line proliferation screening and 12 ovarian cancer patient-derived xenograft (PDX) models *ex vivo*. Consequently, orally administered ACR-2316 was well tolerated and produced robust, dose-dependent tumor growth inhibition in xenograft models [126]. These findings support its advancement toward clinical development as a monotherapy drug.

#### 3.4.3 Debio 0123

Debio 0123, a selective WEE1 Inhibitor, has demonstrated promising antitumor efficacy in preclinical GBM models and exhibits efficient brain penetration. According to Bellon et al., Debio 0123 reduced the IC50 of TMZ and enhanced radiation-induced cell death in primary GBM cell lines *in vitro*. *In vivo* studies showed favorable brain distribution of Debio 0123 in mice, rats, and monkeys, with brain-to-plasma ratios of 0.6, 1.52, and 4, respectively. In orthotopic tumor models, the tumor-to-plasma ratio was found to be 0.62. Also, in GBM xenografts (U87-MG), Debio 0123 suppressed tumor growth by up to 73.7% (brain) and 57.5% (subcutaneous). When combined with TMZ, it induced sustained complete regressions in 75% of mice for up to 100 days, with good tolerability over a 28-day treatment. Additionally, the oral bioavailability of Debio 0123 is pH-dependent, suggesting it should not be taken with proton pump inhibitors (PPIs) [127]. However, food intake had minimal impact on absorption, suggesting that administration with meals would improve patient compliance.

### 3.5 DNA-PK Inhibitors

Inhibiting DNA-PK kinase activity is being investigated as a cancer treatment approach, particularly in conjunction with drugs that induce DNA-DSB140 [41,128]. DNA-PK inhibition is thought to disrupt NHEJ, thereby sensitizing cancer cells to radiation- or chemotherapy-induced DNA damage. There have been numerous SMIs targeting DNA-PK, some of which are currently undergoing clinical trials.

#### 3.5.1 Targeting Ku70/80 Heterodimer for DNA-Pk Inhibition

Targeting KU70/80 heterodimer-DNA interaction is a novel strategy for DNA-Pk suppression, as Ku70/80 is essential for DNA-Pk activation by binding to DNA termini and recruiting DNA-Pk to damage sites [128,129]. Ku-DNA binding inhibitors (Ku-DBis) are designed to prevent this initial step, thereby blocking DNA-Pk catalytic activity, and disrupting the NHEJ pathway and increasing cancer cell sensitivity to DSB-inducing treatments [128,130].

Preclinical studies by Mendoza-Munoz et al. show that Ku-DBis effectively inhibits DNA-Pk and NHEJ with nanomolar activity, sensitizing non-small cell lung cancer (NSCLC) cells to ionizing radiation (IR), bleomycin, and etoposide [128,129]. Ku-DBis enhances DDR suppression by reducing DNA-Pkcs autophosphorylation, activating ATM-p53 signaling, leading to p53 phosphorylation [131]. To improve efficacy, researchers have optimized the structure-activity relationships (SARs) of Ku-DBis by developing oxindole derivatives within the X80 core that enhance cellular uptake, selectivity, and Ku inhibition. These modifications improve inhibition of DNA-DSB repair, reduce nonspecific protein binding, and enable serum-based cellular studies. *In vivo* studies in NSCLC xenograft models also confirmed the therapeutic potential of Ku-DBis, which inhibited IR-induced autophosphorylation of DNA-Pkcs and suppressed tumor cell proliferation and DDR signaling. These advancements mark a significant step toward the clinical application of Ku-DBis for cancer therapies.

#### 3.5.2 SY-7021

SY-7021 is a highly potent and selective DNA-Pki that shows approximately 400-fold selectivity over ATM and ATR. According to investigations by Zhang et al., SY-7021 dose-dependently suppressed cellular NHEJ efficiency in a reporter assay and significantly inhibited cancer cell proliferation both alone and in combination with doxorubicin (DOX) [132]. The SY-7021/DOX combination enhanced phosphorylation of H2AX (Ser139), CHK2 (Tyr68) and p53 (Ser15) and induced G2/M arrest and cell death in MDA-MB-468 cells. *In vivo*, oral administration of SY-7021 twice daily led to dose-dependent tumor growth inhibition in NCI-H1703 xenografts, achieving 105.6% tumor growth inhibition (TGI) at 60 mg/kg without noticeable weight loss. SY-7021 also demonstrated favorable pharmacokinetics and safety profiles [132]. These results support its potential as a promising monotherapy or combinatorial treatment option across multiple cancer types.

#### 3.5.3 Peposertib (M3814A)

Peposertib (M3814) is a potent, specific, orally available DNA-Pki that enhances the efficacy of DNA-damaging agents like topoisomerase II inhibitors and IR [133,134]. It effectively suppresses DNA-Pk catalytic activity, increasing cancer cell sensitivity to DSB-inducing treatments [134]. In preclinical studies, M3814, when combined with IR, induced complete tumor regression in xenograft models without toxicity, highlighting its potential in cancer RT. Combination treatments with peposertib and chemotherapeutics like DOX, epirubicin, and etoposide showed synergistic antiproliferative effects in TNBC cell lines [133]. Moreover, in xenograft models, combination treatments induced tumor regression and triggered pro-inflammatory responses. Additionally, peposertib in combination with IR significantly reduced tumor burden in cervical cancer models, supporting further clinical testing of peposertib with IR [135].

#### 3.5.4 BAY-8400

BAY-8400 originated from a screen aimed at identifying ATRis, during which a triazoloquinoxaline compound was found to inhibit ATR, ATM, and DNA-Pk [136]. This initial hit prompted lead optimization to enhance selectivity and potency toward DNA-Pk, ultimately yielding BAY-8400, a novel and selective DNA-PKi. This development underscores the value of cross-target screening in DDR pathways and highlights the potential of multi-kinase scaffolds [136]. BAY-8400 selectivity inhibits DNA-PK and prevents DSB repair, leading to DNA damage accumulation, genomic instability, and apoptosis, especially in cancer cells that rely heavily on NHEJ due to deficiencies in other repair pathways [137].

Preclinical studies have also demonstrated that BAY-8400 synergizes with DNA-damaging therapies, notably targeted alpha therapy (TAT), which utilizes alpha-emitting radionuclides for radiotherapeutics to induce DSBs. Furthermore, in combination with prostate-specific membrane antigen (PSMA)-targeted thorium-227 conjugates (BAY 2315497), BAY-8400 significantly enhanced antitumor efficacy in prostate cancer xenografts [136]. This effect stems from BAY-8400’s ability to inhibit DNA repair of alpha-particle-induced DSBs, making TAT more lethal to tumor cells [136,138]. BAY-8400 has also been optimized for high selectivity over PI3K and related kinases, minimizing off-target effects and improving its therapeutic window [139]. Related inhibitors, such as AZD7648, similarly showed minimal off-target activity across the kinome, underscoring the importance of DKA-Pk-specific inhibition in clinical development. Related inhibitors like AZD7648 similarly showed minimal off-target activity across the kinome, emphasizing the importance of DKA-Pk-specific inhibition in clinical development [139]. Thus, combining DDRis with DNA-damaging agents such as TAT represents a novel intervention in oncology, as blocking DSB repair sensitizes cancer cells to TAT-induced treatments, enhancing efficacy and potentially allowing dose reductions to mitigate toxicity [136,138].

#### 3.5.5 WNC0901

WNC0901 is a highly potent and selective DNA-Pkcs inhibitor developed by Mladek and colleagues. Mladek et al. reported that WNC0901 exhibits potent kinase inhibition with an IC50 of 0.071 nM in a cell-free system, demonstrating over 30-fold greater selectivity than ATM, ATR, mTOR, and PI3K [140]. In U251 glioma and A549 lung cancer cells, 300 nM WNC0901 combined with 5 Gy radiation effectively suppressed DNA-Pkcs autophosphorylation. In HT29 cells, it showed an IC50 of 32.7 nM following 10 Gy exposure. Clonogenic assays revealed enhanced radio-sensitization, with survival dropping from 10% (radiation alone) to 1.5% at 100 and to 0.04% at 300 nM. Similarly, A549 cells showed reduced survival to 0.2% with combination therapy vs 19% with radiation alone [140]. These findings highlight the potential of WNC0901 as an effective radiosensitizer in GBM and lung cancer by targeting DNA-Pkcs.

In summary, based on data from numerous preclinical studies, DDR can be considered a class of anticancer drugs with the potential to inhibit cancer growth. However, clinical studies are warranted to assess the reproducibility of the preclinical findings to choose the most suitable DDR-based therapeutics to prevent cancer growth.

### 3.6 Challenges of DNA Damage Inhibitors

While the above-mentioned DNA damage inhibitors have demonstrated promising efficacy in targeting tumors with defective DNA repair pathways, numerous preclinical and clinical studies have indicated their potential to cause significant toxicity, especially when combined with chemotherapy. In this context, this section emphasizes the resistance mechanisms and drawbacks associated with DNA damage inhibitors.

#### 3.6.1 ATMi and ATRi

Preclinical research suggests that ATMis can radiosensitize tumor cells, particularly those with deficiencies in the DDR pathway [141]. In colorectal cancer, ATMi has been shown to enhance the radiosensitizing effects of Bragg peak protons more than X-rays or entry protons, suggesting a potential strategy to improve RT [141]. Additionally, combining berzosertib with irinotecan showed promising activity in solid tumors with ATM mutations [142]. The clinical effectiveness of ATMi presents variability while researchers examine resistance mechanisms. A resistance mechanism activates ATM when ATR inhibitors are present, leading to G1 cell cycle arrest and reduced cytotoxicity.

The simultaneous inhibition of ATM and ATR proteins results in increased therapeutic effectiveness, according to Turchick et al. [36]. Furthermore, ATM-knockout (ATM-KO) prostate cancer cells can still repair IR-induced DNA damage through ATR and DNA-PKcs activation, highlighting the need for dual inhibition [143]. Overcoming ATM resistance involves combining ATM with ATR or DNA-PKis or targeting compensatory pathways [143–145].

Similarly, ATRi resistance arises through mechanisms such as enhanced replication fork progression, [146] cyclin C/CDK8 inhibition [147], or ATM pathway activation [36]. Combining ATRi with other DDRis such as ATMi and WEE1, along with biomarker-driven targeting of bypass pathways, offers a strategy to counteract resistance [52,146].

#### 3.6.2 CHK1i

The use of CHK1i is justified because cancer cells, which often have impaired G1/S checkpoints, depend primarily on the CHK1-mediated G2/M checkpoint to survive. Clinical trials with CHK1i have demonstrated inconsistent efficacy despite encouraging preclinical results, and resistance mechanisms remain a significant obstacle. Numerous resistance mechanisms to CHK1i have been identified, including alterations in the thioredoxin system [148], PI3K/AKT signaling bypass [144], and increased WEE1 levels [149]. Studies also show a substantial correlation between the degree of acquired resistance and the messenger RNA (mRNA), protein levels, and DNA copy number of Wee1 [149]. Nevertheless, a combination of CHKi and PI3K/mTOR inhibitors has been shown to increase DNA damage, mitotic catastrophe, and attenuate cell viability [150].

#### 3.6.3 WEE1i

WEE1i resistance has been shown to occur through the overexpression of MYT1 kinase, which blocks WEE1 by activating CDK1 [151]. This results in shortened premature mitotic entry and decreased mitotic duration, leading to enhanced survival rates (%) of adavosertib-treated cells [151]. This upregulation of MYT1 may also influence resistance to ATRi and CHK1i. Therefore, combining WEE1i with CHK1i ATRi, and Bcl-2 homology domain 3 (BH3) mimetics may be a useful strategy to inhibit treatment resistance [123]. Additionally, ZN-d5, as well as other BH3 mimetics, has been reported to cause caspase-induced DNA damage, along with Wee1 and ribonucleoside diphosphate reductase subunit M2 (RRM2) degradation, leading to synergistic efficacy when combined with ZN-c3 [123].

#### 3.6.4 DNA-Pki

Despite the therapeutic potential of DNA-Pkis, resistance remains a significant hurdle. Intrinsic resistance can arise from tumor-specific genetic alterations or activation of alternative DNA repair pathways [96,152]. Acquired resistance may develop over time as cancer cells evolve in response to the selective stress. Notably, TP53-deficient tumors exhibit an increased reliance on MMEJ, conferring tolerance to DNA-PK inhibition [153]. In such cases, combining DNA-Pki with MMEJ (Polθ) inhibitors may induce synthetic lethality. Inhibition of DNA-PK has also been shown to enhance Polθ expression and end resection, promoting MMEJ activity and limiting the efficacy of monotherapy [153]. Additionally, normal tissues when exposed to DNA-Pki develop increased sensitivity to DNA-damaging agents like radiation and chemotherapy which prompts toxicity concerns [154]. The clinical translation of this approach demands the enhancement of the therapeutic index through increased tumor-selective cytotoxicity combined with reduced harm to normal tissues.

Across DDR-targeting agents, clinical outcomes remain heterogeneous and highly context-dependent. In pivotal early-phase studies, the ATR inhibitor BAY1895344 combined with topotecan achieved a median progression-free survival (PFS) of 9.2 months compared with 3.4 months with chemotherapy alone, while the WEE1 inhibitor adavosertib produced objective response rates (ORRs) of 26–38% in CCNE1-amplified tumors and platinum-resistant ovarian cancer [155,156]. Another phase I/II multi-tumor study using the ATR inhibitor ceralasertib (AZD6738) in ATM-deficient solid tumors and hematologic malignancies reported durable responses, with median overall survival (OS) approaching 12 months in select cohorts [157]. For PARP inhibitors, large-scale trials continue to demonstrate a survival benefit in BRCA-mutated ovarian and breast cancers, with median PFS improvements of 3–7 months compared with chemotherapy [158]. Although the therapeutic efficacy of DDR-targeting agents has shown great potential, class-related toxicities remain a significant limitation. For example, grade ≥ 3 hematologic adverse events (anemia, neutropenia, thrombocytopenia) have been reported in 25–45% of patients, alongside fatigue, nausea, and gastrointestinal intolerance [159]. More recently, Romesser et al. conducted a Phase Ib study of the DNA-PK inhibitor peposertib combined with neoadjuvant chemoradiation therapy (CRT) in patients with locally advanced rectal cancer (LARC). The patients were treated for 5 to 5.5 weeks with 50–250-mg peposertib administered once daily, capecitabine at 825 mg/m^2^ administered twice daily, and radiotherapy (RT) administered 5 days per week. In summary, while peposertib acts as a potent radiosensitizer, this Phase Ib study did not demonstrate clinical benefit in LARC patients. It was limited by significant gastrointestinal toxicity, highlighting challenges in combining DNA-PK inhibitors with full-dose chemoradiation [160]. Furthermore, intermittent or sequential dosing schedules, biomarker-guided patient selection (e.g., HRD or SLFN11 positivity), and nanoparticle-based formulations are now being explored to widen the therapeutic index and reduce overlapping myelosuppression in combination regimens [97].

## 4 Tumor Microenvironment-Induced Resistance to DDR Inhibitors and Immunologic Remodeling for Enhanced Effectiveness

**Figure 3:**
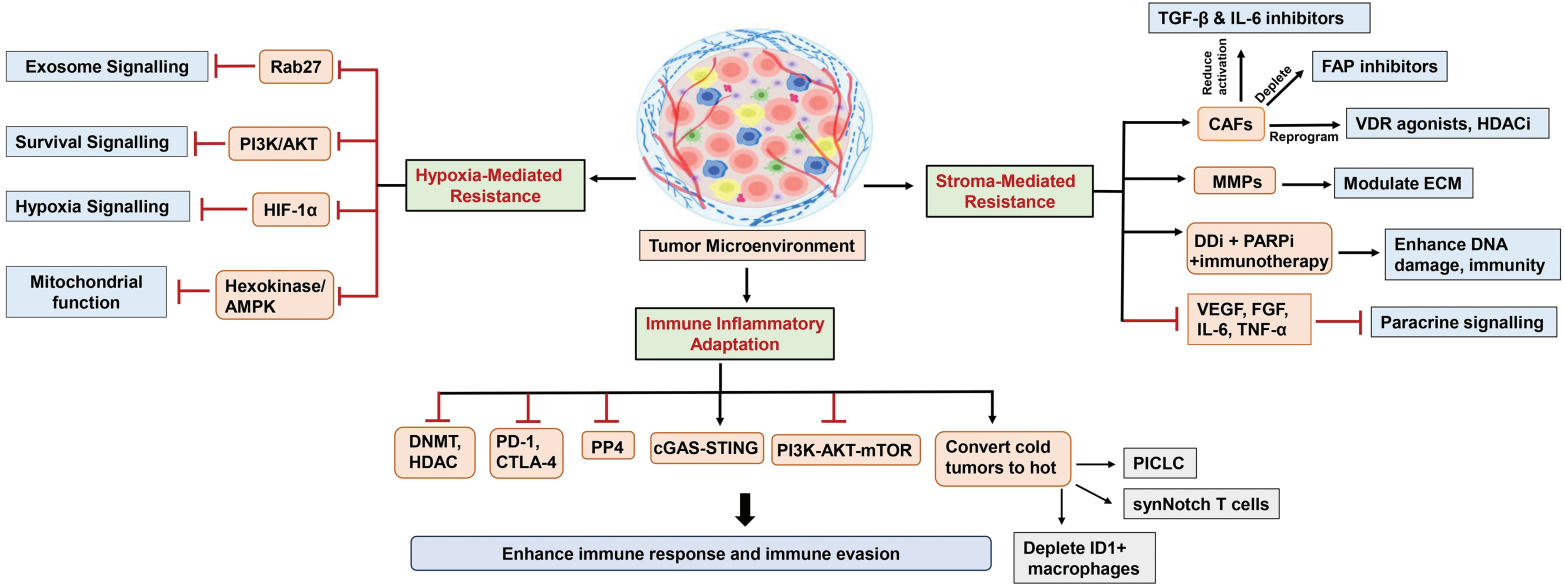
Schematic representation of mechanisms of TME-induced resistance to DNA damage response therapies

The tumor microenvironment (TME) exerts decisive control over the efficacy of DDRis, functioning as both a refuge for resistant tumor cells and a dynamic regulator of treatment-induced immunity. Hypoxia, oxidative stress, and stromal remodeling activate compensatory repair pathways that sustain survival under genotoxic stress. At the same time, inflammatory cues and immune cell plasticity determine whether DDR perturbation triggers effective antitumor immunity or immune escape. Interactions among fibroblasts, endothelial cells, macrophages, and lymphocytes continuously reshape local cytokine gradients, vascular permeability, and redox balance factors that collectively dictate DDRi penetration and response. Understanding how these stromal, metabolic, and immune components intersect is essential for converting DDRi-induced stress into durable therapeutic benefit. The following subsections examine how hypoxia, stromal signaling, inflammation, and immune remodeling converge to create resistance, and how selective DDR targeting can reprogram the TME toward a more immunostimulatory state (Fig. 3).

### 4.1 Hypoxia-Induced Resistance to DDR Inhibitors

Hypoxia, commonly found in the TME of solid tumors due to rapid growth and abnormal vasculature, drives therapy resistance by altering the metabolism and gene expression of cancer cells [161,162]. Under low-oxygen conditions, cancer cells activate hypoxia-inducible factors (HIFs), which drive adaptive mechanisms that support their survival, facilitate DNA repair, and enable them to evade treatment [163,164]. The activation of HIF-1α leads to the upregulation of DNA repair pathways, making tumor cells more resilient to genotoxic stress [165].

HIF-1 upregulates DNA repair genes, thereby enhancing repair capacity and reducing sensitivity to drugs such as PARP, ATM/ATR, and CHK1/CHK2 inhibitors [166,167]. It also disrupts cell cycle checkpoints and promotes epithelial-mesenchymal transition (EMT), thereby further enhancing resistance [167,168]. Additionally, hypoxia affects mitochondrial dynamics and promotes exosome-mediated signaling that spreads resistance traits [169,170]. In addition, hypoxia impairs drug delivery by disrupting vascular function and altering the tumor’s metabolic environment [171,172]. It also promotes the expansion of cancer stem-like cells and evasion of cell death by inducing autophagy and upregulating survival proteins [169,173,174].

A key resistance mechanism involves the hypoxia-induced upregulation of DDIT4 (also known as REDD1), which inhibits mTORC1 signaling, thereby aiding cell survival under stress and reducing sensitivity to radiotherapy and temozolomide. Whereas suppression of DDIT4 (REDD1) restores sensitivity [161]. Metabolic reprogramming in the TME plays a vital role in driving tumor progression by mediating interactions among cancer cells, fibroblasts, and immune cells [162]. Key regulators include mTOR, a downstream effector of PI3K/Akt signaling that controls cell growth, survival, and metabolism [175], and tAMPK, a central energy sensor that maintains metabolic balance [176].

The hypoxia-related gene COL5A1 is linked with poor prognosis while regulating immune pathways, which may affect chemotherapy response [162]. Moreover, hypoxic conditions promote genomic instability, which boosts mutation rates and develops resistance to DDRis [177]. Specific resistance mechanisms include enhanced homologous recombination under hypoxia, which undermines PARP inhibition, and HIF-1-driven bypass of ATM/ATR checkpoint signaling. CHK1/CHK2 inhibitor resistance arises through compensatory pathways and checkpoint regulation [167].

### 4.2 Stromal Interaction and DDR Inhibitors Resistance

Stromal cells, including fibroblasts, endothelial cells, and pericytes, are integral components of the TME, influencing resistance to DDRis through growth factor secretion, extracellular matrix (ECM) remodeling, immune modulation, and impaired drug delivery [178]. Among them, cancer-associated fibroblasts (CAFs) are particularly prominent. They secrete factors such as HGF and EGF that activate survival pathways, while their ECM remodeling creates a dense, drug-impermeable barrier [178].

CAFs also secrete cytokines such as transforming growth factor beta (TGF-β) and interleukin 6 (IL-6), which activate the PI3K/Akt, MAPK, and STAT3 pathways, thereby enhancing tumor cell survival and reducing the sensitivity of DDRis [179,180]. TGF-β promotes EMT, while IL-6 upregulates anti-apoptotic proteins, contributing to chemoresistance [181]. Additionally, CAF-derived ECM proteins, such as collagen, hinder drug penetration and sequester pro-survival cytokines [180,182]. Immunologically, CAFs recruit immunosuppressive cells, such as myeloid-derived suppressor cells (MDSCs) and regulatory T cells (Tregs), which suppress the activity of cytotoxic T cells and natural killer cells, thereby diminishing the immune-mediated response to DDRis [183–185].

Paracrine signaling from stromal cells further enhances resistance by activating pro-survival pathways in tumor cells. Growth factors like VEGF and FGF engage receptor tyrosine kinases (RTKs), promoting angiogenesis, ECM remodeling, and DNA repair while inhibiting apoptosis [179]. Cytokines, including IL-6 and TNF-α, activate inflammatory signaling pathways via STAT3 and NF-κB, which fosters genomic instability and drug resistance [186]. Stromal-derived exosomes also transfer resistance-promoting molecules such as microRNAs, proteins, and ECM components that bolster tumor survival and block drug penetration [187,188].

### 4.3 Inflammatory Signaling, Immune Cell Infiltration, and DDR Inhibitor Sensitivity

Inflammatory signaling in the TME significantly affects the response to DDRis [189]. Chronic inflammation supports tumor growth, angiogenesis, metastasis, and therapy resistance by creating a pro-survival environment. Pathways such as NF-κB and STAT3 upregulate anti-apoptotic proteins and DNA repair enzymes, thereby reducing the effectiveness of DDRis [189]. Since DNA damage itself activates NF-κB, DDR inhibition may amplify its signaling, promoting resistance to therapies that depend on inducing DNA damage [190]. Immune cell infiltration in the TME is essential for modulating sensitivity to DDRis [178,191]. DDRis increase tumor immunogenicity by elevating DNA damage, thereby enhancing the recruitment and activation of immune cells [189]. Nonetheless, immunosuppressive cells, such as myeloid-derived suppressor cells (MDSCs) and Tregs, can counteract this effect, promoting resistance [178].

Ultimately, the balance between immune activation and suppression in the TME determines the response to DDRis. Tumors with high cytotoxic immune infiltration and low immunosuppression respond better, while those dominated by suppressive cells are more resistant [178,189]. Combining DDRis with immunotherapies such as checkpoint blockade may improve therapeutic outcomes [191].

### 4.4 Immune Plasticity and DDR-Driven Remodeling of the Tumor Microenvironment

Inhibition of the DDR induces complex remodeling of the tumor immune landscape [192]. Myeloid and lymphoid populations adapt dynamically to DNA-damage-associated stress signals, balancing cytotoxic and suppressive functions [193]. For instance, tumor-associated macrophages (TAMs) exhibit remarkable plasticity, transitioning between pro-inflammatory (M1-like) and immunosuppressive (M2-like) states in response to signals from the TME, including cytokine cues (IL-12, IL-23, IL-4, IL-10, and IL-13) and ROS, which can be altered by DDR-targeting therapies [194]. Recent studies demonstrate that activation of the cGAS/STING pathway by accumulation of cytosolic DNA fragments following DDR inhibition in tumor or innate immune cells triggers type I interferon (such as IFN-β) release and chemokine production (CXCL8, CCL2), which contributes to both inflammation and recruitment of monocytes/macrophages into the TME, where additional cytokines direct their polarization [195,196]. However, accumulating evidence also suggests that chronic activation of the cGAS/STING pathway may exert an antitumor or pro-tumor effect, depending on numerous factors such as the rate of progression and the affected tissue [196,197]. Moreover, studies have reported an effect of DDR-targeted agents on immune cells such as Tregs and myeloid-derived suppressor cells (MDSCs) with immunosuppressive functions [194]. These context-dependent outcomes underscore that DDRi therapy can both ignite and dampen immunity, depending on exposure timing, tissue hypoxia, and stromal composition. Mechanistic dissection of this plasticity is therefore critical for identifying optimal sequencing with checkpoint blockade or cytokine modulation.

### 4.5 Mechanisms of “Cold-to-Hot” Tumor Conversion and Therapeutic Integration

In recent years, DDR-targeted therapies have emerged as potent activators of immunologic cell death (ICD) via enhancing tumor immunogenicity and activating tumor-specific immune sensing pathways (e.g., cGAS/STING-IFN), linking genotoxic stress to tumor-specific immune responses [198,199]. Inhibition of PARP, ATR, or WEE1 leads to accumulation of cytosolic DNA fragments and micronuclei, activating cGAS-STING-TBK1-IRF3 signaling and driving type I interferon and antigen-presentation pathways [200]. Cells have complex damage-associated molecular pathways that work harmoniously under stress or injury. As cancer cells die immunogenically, they release damage-associated molecular patterns (DAMPs) such as HMGB1, ATP and surface-exposed calreticulin [201–203]. These signals promote dendritic cell recruitment and the presentation of tumor-specific antigens to T cells, thereby inducing an immune response [203]. These processes together promote immunologically “cold” tumors into “hot,” inflamed lesions. However, immune escape via secondary up-regulation of programmed death-ligand 1 (PD-L1) and secretion of TGF-β or IL-6 can re-establish suppression, highlighting the importance of rational combinations [204,205]. For instance, studies using experimental preclinical models have demonstrated the favorable effects of DDR inhibitors when combined with immune checkpoint blockade, further enhancing the cytotoxic T-cell infiltration and tumor regression [206]. Additionally, targeting of stromal or angiogenic pathways (anti-TGF-β, anti-VEGF) can further stabilize immune activation. While preclinical studies have shown benefits, the overall success of the above-described strategies depends on several factors, including careful scheduling to exploit the early window of DAMP-driven stimulation before feedback inhibition predominates. Ultimately, integrating DDR modulation with immunotherapy offers a mechanistic route to overcoming TME-induced resistance and achieving durable “cold-to-hot” tumor reprogramming.

The impact of DDR modulation on the TME varies across tumor types, mutational landscapes, and baseline immune contexture [207]. Tumors with pre-existing interferon signaling or high neoantigen load, such as BRCA-mutated or mismatch-repair-deficient cancers, are especially susceptible to DDRi-driven immune activation. This susceptibility is linked to increased tumor mutation burden, neoantigen production, and heightened interferon pathway activity, collectively enhancing tumor immunogenicity and responsiveness to immunotherapy combined with DDR-targeted agents [208]. In contrast, hypoxic or desmoplastic tumors often remain refractory because of impaired drug penetration and persistent immunosuppressive signaling [209]. These tumors feature hypoxic regions that could induce genetic reprogramming and alter the DDR pathway, subsequently leading to resistance to DDR inhibitors and other therapies [210]. Also, persistent hypoxia in TME induces HIF-1 expression, which further promotes immunosuppression and reduces infiltration and function of immune effector cells [209,210].

While there have been advances in DDR-targeted therapies, these have not been without their limitations and challenges. In Section 5, we discuss advancements in nanocarrier-based delivery systems that may help overcome these barriers by improving intratumoral accumulation and reducing systemic toxicity, thereby enabling more effective immunotherapy combinations. Overall, DDRi-induced reprogramming of the TME reflects a dynamic balance between immunoregulatory mechanisms. Clarifying the temporal sequence of these responses, early DAMP-mediated activation followed by compensatory checkpoint induction, will be essential for optimizing dosing schedules and identifying biomarkers of durable “cold-to-hot” conversion. As this mechanistic insight deepens, DDR-targeted therapy is poised to emerge as a central modulator linking DNA repair dynamics to coordinated antitumor immunity.

### 4.6 Potential Strategies to Overcome TME-Induced Resistance

Overcoming TME-induced resistance to DDRis requires a comprehensive strategy targeting both cancer cells and their microenvironment [211,212]. Approaches include combining DDRis with chemotherapy, radiotherapy, immunotherapy, or agents like CHK1 or ATRis to enhance DNA damage and bypass resistance mechanisms [101,167,213]. In this section, we provide a detailed breakdown of current and emerging approaches to overcome TME-induced resistance.

#### 4.6.1 Overcoming Hypoxia-Mediated DDR Inhibitor Resistance

Vasculature normalization using anti-angiogenic agents (e.g., bevacizumab) can reduce hypoxia and improve treatment delivery [161]. Targeting cancer metabolism, such as inhibiting pyrimidine synthesis, sensitizes cells to genotoxic agents [214]. Hypoxia-activated prodrugs (e.g., tirapazamine) selectively target low-oxygen environments [215], while HIF-1α pathway inhibition disrupts hypoxia-mediated signaling [165,216]. Improving oxygen delivery through vascular normalization, hyperbaric oxygen therapy, or the use of platinum-based nanoparticles can help reverse resistance [164,172,215,217]. Combining DDRis with chemotherapy, radiotherapy, or immunotherapy enhances efficacy in hypoxic tumors [172,216]. Importantly, combining DDRis with hypoxia-targeting agents (e.g., HIF-1 inhibitors or vascular normalization therapies) has shown promise [218,219]. Inhibiting the PI3K/AKT/HIF-1 axis or blocking exosome communication can also restore sensitivity [166,170]. Moreover, integrating DDRis with immunotherapy may overcome hypoxia-induced immunosuppression and improve outcomes [219]. Additionally, disrupting metabolic dependencies, such as glycolysis and mitochondrial function, also sensitizes these tumors [220,221].

Targeting the TME by inhibiting lysyl oxidase (LOX) or extracellular vesicle (EV) signaling improves drug delivery and reduces chemoresistance [220,222]. Hypoxia profiling and imaging technologies, such as photoacoustic imaging (PAI), can further guide precision treatment [223]. Clinical studies are evaluating hypoxia-targeted therapies to overcome resistance [224].

#### 4.6.2 Overcoming Stromal-Mediated DDR Inhibitor Resistance

Effective treatment of stromal-mediated DDRi resistance demands simultaneous targeting of both tumor cells and stromal components. Practical approaches involve CAF targeting, ECM modulation, paracrine signaling inhibition, and the combined use of DDRis with additional treatments. Targeting the protective role of CAF enhances the efficacy of DDRi. This includes inhibiting CAF activation with TGF-β and IL-6 inhibitors (e.g., galunisertib, tocilizumab), depleting CAFs with FAP inhibitors such as talabostat, or reprogramming CAFs using VDR agonists or histone deacetylase inhibitors (HDACis) [182,185]. Also, modulating the ECM improves drug delivery and reduces resistance. Approaches include degrading collagen with collagenase [182], inhibiting LOX with BAPN [181], and disrupting ECM-integrin interactions using cilengitide [181].

Furthermore, inhibiting paracrine signaling can block protective signals, making tumor cells more susceptible to DDRis. Strategies include targeting growth factors (e.g., VEGF, FGF), cytokines (e.g., IL-6, TNF-α), and exosomes to enhance therapy sensitivity [179,187]. Combining DDRis with chemotherapy, radiation, or immunotherapy enhances treatment outcomes. PARPis (e.g., olaparib) and radiation therapy increase [225] DNA damage, while DDRis in immunotherapy, enhance the immune response by boosting antigen expression [211,226]. These strategies highlight the importance of combination therapies and TME targeting to overcome stromal-mediated resistance and improve cancer treatment outcomes [227].

#### 4.6.3 Combination with Immune Checkpoint Inhibitors (ICIs)

DDRis can increase PD-L1 expression and tumor immunogenicity, making tumor cells more susceptible to ICIs. This synergy has shown promise in preclinical and clinical studies [228]. Raja et al. found that inhibiting protein phosphatase 4 (PP4) with carboplatin activates NF-κB and STAT1, thereby enhancing proinflammatory cytokine production and improving type I interferon responses, which suggests improved DDR inhibitor sensitivity through immune activation [189]. These pathways are interconnected, allowing NF-κB to trigger STAT3 through cytokine release, forming a feedback loop that maintains inflammation and promotes immune evasion by upregulating checkpoints, such as PD-L1 [229]. Therefore, targeting multiple inflammatory signals may be essential to enhance DDRi efficacy [189].

#### 4.6.4 Enhancing Immune Infiltration into Cold Tumors

PP4 inhibition, achieved with fostriecin, combined with carboplatin, has been shown to enhance DNA damage and inflammatory signaling, resulting in increased infiltration of CD8+ T cells and NK cells and reduced tumor growth in ovarian cancer models [189]. This highlights PP4 as a target to enhance anti-tumor immunity, which targets interferon (IFN) responses and immune cell recruitment [191]. While DDRis can enhance this effect, chronic IFN signaling in some tumors may drive immunosuppression through the upregulation of checkpoint proteins, such as PD-L1 [229]. Approaches to convert cold tumors to hot tumors include 1) PICLC (polyinosinic-polycytidylic acid stabilized with poly-lysine and carboxymethylcellulose) to promote CD8+ T cell trafficking and activation, 2) localized delivery of IL-2 via engineered synNotch T cells to trigger localized immune activation, and 3) depleting ID1+ macrophages, with blocking CD8+ T cell infiltration, enhances antitumor immunity [230].

#### 4.6.5 Activating the cGAS-STING Pathway

cGAS-STING agonists are being explored as adjuvants to overcome immune exclusion. For instance, the simultaneous use of DDRis and cGAS-STING activators enhances antitumor immune responses by augmenting type I interferon signaling, increasing cytoplasmic DNA levels, and improving T-cell infiltration, thereby overcoming resistance in BRCA1-deficient tumors [231]. Moreover, ATRi AZD6738, combined with radiotherapy and anti-PD-L1 treatment, has been shown to boost CD8^+^ T-cell activation while decreasing immunosuppressive populations and promoting long-term immune memory via cGAS-STING signaling pathways [232,233]. Additionally, STING agonists transform TAMs into anti-tumor cells, thereby restoring PARPi effectiveness through host STING-dependent type I IFN production and boosting CD8^+^ T-cell activity [234]. Furthermore, inhibition of PARP or CHK1 in SCLC has been shown to increase PD-L1 and chemokine levels through the cGAS-STING-TBK1-IRF3 pathway, making tumors more responsive to immune checkpoint blockade [235].

Similarly, the combination of PARPi with radiotherapy and anti-PD-L1 treatment stimulates cGAS-STING pathways while enhancing chemokine output and T-cell infiltration in SCLC, leading to improved antitumor results [236]. The combination of the CDC7 inhibitor XL413 and PARPi in ovarian cancer models has also been shown to enhance replication stress while activating cGAS–STING–mediated interferon responses, leading to significant tumor regression [237]. These findings collectively demonstrate the potential to improve cancer immunotherapy outcomes by activating innate immunity via DDR-induced cGAS-STING signaling. Therefore, the combination of cGAS-STING agonists and DDRis may provide new insights for treatment [235,238].

#### 4.6.6 Inhibiting the PI3K-AKT-mTOR Pathway

Aberrant activation of the PI3K-AKT-mTOR Pathway contributes to immune evasion. Its inhibition can restore immune infiltration and sensitize tumors to ICIs, particularly those with PI3K pathway mutations [239].

#### 4.6.7 Epigenetic Modulation

Agents such as DNA methyltransferase inhibitors (DNMTis) and HDACis can enhance antigen presentation, increase T cell recruitment, and reactivate exhausted immune cells, making tumors more responsive to DDRis [228,240].

Overall, further research must focus on multi-pronged strategies that disrupt both the intrinsic survival mechanisms of tumor cells and the protective effects of the TME. Furthermore, future success will depend on rational trial designs, biomarker-driven patient selection, and continued mechanistic exploration.

## 5 Recent Advancements in Enhancing DDR Inhibitor Efficacy

**Figure 4:**
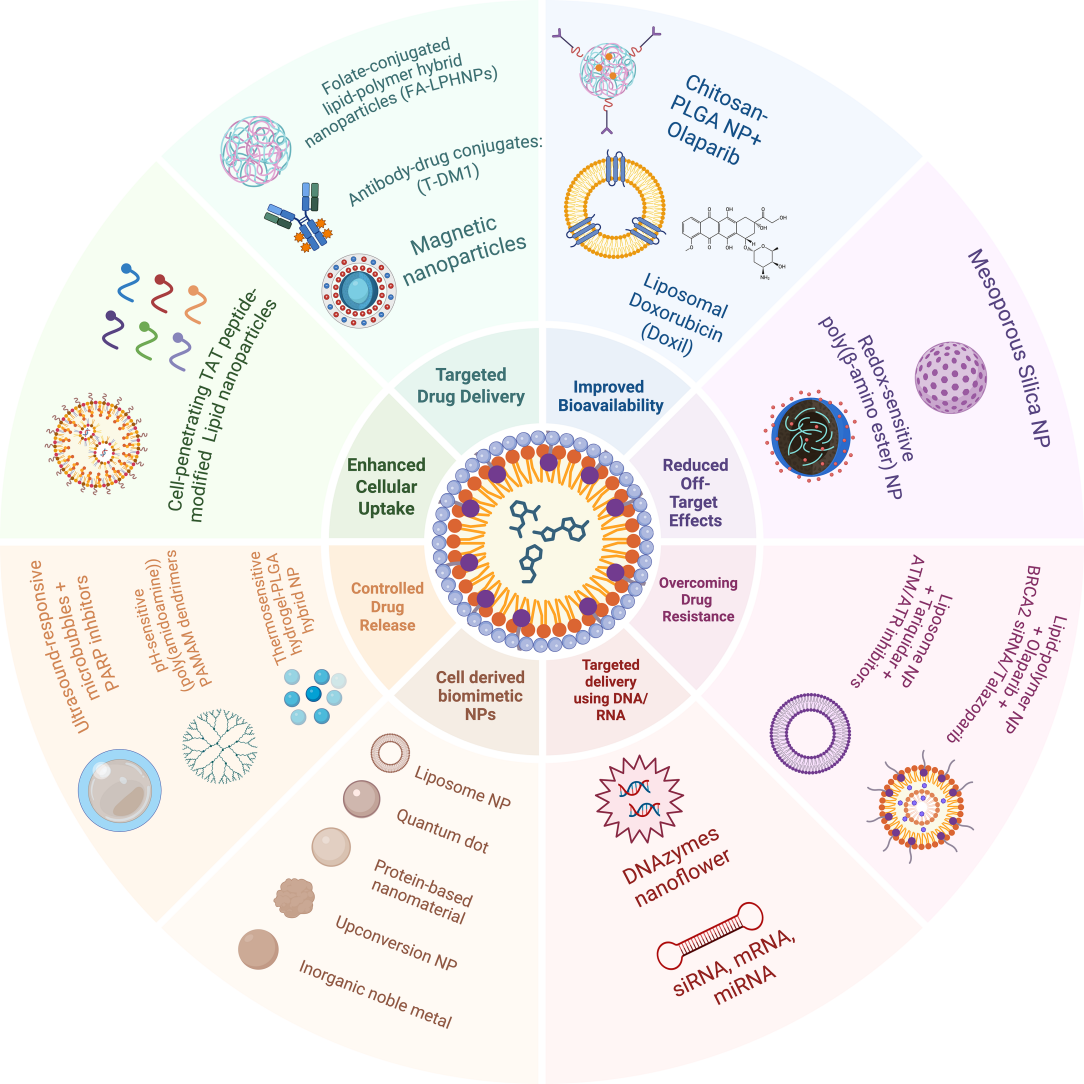
Potential application of nanoparticle-mediated drug delivery to enhance treatment responses to DNA damage response inhibitor therapies

The above-described studies indicate that while DNA damage inhibitors attenuate cancer growth, they have not been without their limitations. In this section, we explore the innovative use of nanoencapsulation techniques (Fig. 4) that enhance the delivery and efficacy of the DDR inhibitor.

### 5.1 Nanoparticle-Based Drug Delivery

Nanoparticle-based drug delivery systems enhance therapeutic precision through diverse platforms, such as liposomes, like Doxil^®^, which leverage the enhanced permeability and retention (EPR) effect for tumor accumulation [241]. For instance, folate-conjugated lipid-polymer hybrids (FA-LPHNPs) have been shown to target cancer cells via receptor-mediated endocytosis [242]. Also, stimuli-responsive systems, such as pH-sensitive mesoporous silica nanoparticles (MSNs), exhibit drug selectivity in the acidic TME [243–245]. Polymeric nanoparticles (e.g., PEG-PLA) have also been shown to improve the bioavailability of hydrophobic drugs, such as PARPis, by achieving sustained release [246–248]. Emerging innovations also include hybrid lipid-polymer nanoparticles (HLPNPs) for dual-drug delivery and biomimetic exosome-mimetic vesicles for enhanced glioblastoma targeting [249]. However, challenges persist in scalability, immune recognition, and long-term safety. Nevertheless, new interventions such as AI-driven drug design and CRISPR-loaded gold nanoparticles promise a breakthrough in personalized therapy [250–252]. Moreover, advances in sustainable nanomaterials and in organ-on-a-chip evaluation aim to address environmental and translational hurdles alongside DDRi interventions [253,254]. As discussed, the clinical success of DDRis targeting PARP, ATM, ATR, and CHK1 is often hindered by issues such as poor pharmacokinetics, limited tumor accumulation, synthetic lethality, and acquired drug resistance. Nanocarriers provide a versatile platform for overcoming these challenges by enhancing drug delivery, improving bioavailability, increasing specificity, and facilitating intracellular uptake. Below are representative studies on the strategies (Fig. 4) by which nanocarrier systems enhance the efficacy of DDRis.

### 5.2 Biocompatibility & Safety

Biocompatible nanocarriers reduce toxicity by utilizing degradable materials and employing surface modification techniques. FDA-approved PLGA-PEG NPs have demonstrated enhanced biocompatibility and biodegradability with several drugs in preclinical and clinical studies over the years, owing to their ability to degrade into CO_2_ and water. PEG minimizes immune recognition while biodegradable lipids like DSPC metabolize into non-toxic fatty acids. Clinical advancements include FDA-approved PLGA-PEG nanoparticles for leuprolide delivery, which degrade into CO_2_ and H_2_O [255], and albumin-bound paclitaxel (abraxane), which utilizes endogenous albumin to reduce immunogenicity in pancreatic cancer [256].

### 5.3 Targeted Drug Delivery

Targeted drug delivery uses tumor-specific ligands or antibodies to direct nanocarriers to receptors overexpressed on cancer cells, thereby minimizing off-target effects. A study by Gu et al. developed folate-conjugated, indocyanine green-loaded lipid-polymer hybrid nanoparticles (FA-LPHNPs) that bind folate receptors (FRs) on ovarian cancer cells, achieving 12-fold higher tumor accumulation in murine models compared to non-targeted systems [257,258]. These nanoparticles exploit receptor-mediated endocytosis, where FR binding triggers internalization, followed by liposomal acidification, which releases cisplatin directly into the cytoplasm, thereby bypassing efflux pumps. Over the years and recently, studies have demonstrated the clinical success of ADCs, such as trastuzumab-emtansine (T-DM1) (anti-HER2), in HER2+ breast cancer [259,260]. As well as the use of magnetic nanoparticles guided by external fields for tumor targeting, underscoring the versatility of ligand-mediated strategies [261,262].

### 5.4 Improved Bioavailability

Nanocarriers enhance the bioavailability of hydrophobic DDRis by shielding them from enzymatic degradation and rapid clearance. Anwer et al. demonstrated that Chitosan-coated PLGA nanoparticles encapsulating olaparib, which increased oral bioavailability by 4.75-fold in preclinical models [263]. PEGylation reduces opsonization by the mononuclear phagocyte system (MPS), while the PLA matrix enables sustained release, maintaining therapeutic plasma levels for 24 h. Studies have also highlighted similar improvements with liposomal doxorubicin (Doxil), where PEGylation extends the circulation half-life to 55 h [264], and a micellar SN-38 formulation that enhances solubility and reduces gastrointestinal toxicity [265,266].

### 5.5 Reduced Off-Target Effects

Stimuli-responsive nanocarrier minimizing off-target effects by releasing drugs selectively in the TME. The application of MSNs may offer an advanced strategy when encapsulated with DDRis, as the hydrazone bond grafted to the MSN’s surface hydrolyzes in acidic conditions, ensuring localized drug release [267]. Some recent advances include the formulation of doxorubicin-loaded redox-sensitive poly (β-amino ester) nanoparticles that degrade in high-glutathione environments to treat breast cancers [268], and MMP-2-expressed nano capsules for colorectal cancer, demonstrating the broad applicability of microenvironment-triggered systems [269].

### 5.6 Enhanced Cellular Uptake

Nanocarriers enhance the cellular uptake of DDRis via receptor-or peptide-mediated mechanisms. An early study by Futaki et al. highlights the role of lipid nanoparticles modified with cell-penetrating TAT peptides, which have been shown to achieve energy-independent enhanced internalization in breast cancer cells [270]. Consequently, a recent study shows that the PLGA-PEG shielded TAT peptide, when exposed only after selective binding to an angiotensin-converting enzyme (ACE2) inhibitor, is exposed and shows increased cellular uptake [271]. Moreover, siRNA-loaded cationic lipid carriers have also shown improved drug uptake in lung adenocarcinoma due to their ability to exploit electrostatic interactions for enhanced drug delivery to treat [272].

### 5.7 Controlled Drug Release

Controlled release systems maintain therapeutic drug levels while minimizing toxicity. For instance, thermosensitive hydrogel-PLGA hybrid (micro-TMZ@PLGA-PEG-PLGA) has been shown to be effective in the sustained release of TMZ in glioblastomas [273]. The hydrogel liquefies at body temperature, allowing PLGA to degrade via hydrolysis into lactic and glycolic acids, which govern zero-order release kinetics [274]. Innovations such as pH-sensitive (poly(amidoamine) PAMAM dendrimers for cisplatin delivery and ultrasound-responsive microbubbles for localized drug release further highlight the potential of programmable nanocarriers to sustain DDR inhibition [275,276].

### 5.8 Overcoming Drug Resistance

Co-delivery nanocarriers address resistance by simultaneously targeting DDR pathways and compensatory mechanisms. While previous investigations report the use of PARP siRNA-loaded lipidoid NPS in treating BRCA-resistant ovarian mice models [277]. Talazoparib-loaded solid lipid NPs (SLNPs) have been reported to directly overcome PARPi resistance in TNBCs that include BRCA-deficient as well as talazoparib-resistant tumor models [278]. The siRNA silences BRCA2 reversion mutations, turning off HR repair, while talazoparib blocks PARP-mediated BER, inducing synthetic lethality. Preclinical studies have also demonstrated similar success with liposome co-encapsulating paclitaxel and the P-gp inhibitor tariquidar, to circumvent efflux pump resistance, as well as dual ATM/ATR inhibitor NPs for TP53-mutant tumors [36,279].

### 5.9 Translational and Regulatory Challenges of Nanocarrier-Based DDR Inhibitor Delivery

While advances in the nanocarrier systems have transformed drug delivery by improving solubility, stability, and targeted accumulation, translating these constructs for DDRis remains complex. Accordingly, there are major obstacles in the clinical application of the nanocarrier systems, including variability in synthesis, limited tumor penetration, and evolving regulatory frameworks. A realistic appraisal of these challenges is essential to ensure that nanocarrier development advances in tandem with pharmacologic and biological understanding.

#### 5.9.1 Manufacturing Scalability and Reproducibility

Recent studies have demonstrated major instabilities in nanoparticle formulations of DDRis, such as variability in size, surface charge (zeta potential), and drug payload loading, each of these factors impacts biodistribution and therapeutic index [280]. Additionally, studies have reported that alterations in size and charge affect tissue penetration, cellular uptake, and clearance rates, which subsequently impact the efficacy and toxicity profiles of DDRis [281,282]. Moreover, differences in solvent composition, mixing energy, or polymer molecular weight can shift key formulation attributes [283]. The above-mentioned discrepancies are often reported while transitioning from small laboratory scale-up process towards manufacturing. For instance, the scale-up process introduces new sources of variability in mixing, material handling, and process control, leading to batch-to-batch inconsistencies that directly impact nanoparticle characteristics critical for biodistribution and therapeutic efficacy [284,285]. Hence, adaptation of synthesis and stabilization protocols, well-optimized analytical methods, such as reverse-phase (RP) HPLC with dynamic light scattering, might enhance reproducibility and regulatory compliance could help to resolve a few concerns [286,287]. Moreover, for DDRi formulations, synchronizing drug release kinetics with transient DDR activation may also help.

#### 5.9.2 Biodistribution, Tumor Delivery, and Biological Barriers

Another challenge in the nanocarrier system is the *in vivo* biological barriers [288]. For instance, hepatic and splenic macrophages mediated uptake often limits their tumor accumulation [289,290]. Additionally, the EPR effect observed in rodent tumor models is inconsistent in human tumors [291]. Therefore, surface modifications such as PEGylation or biomimetic coatings can prolong circulation and reduce immune recognition but may also impede cellular uptake or endosomal escape [292,293]. Quantitative imaging including advanced particle tracking with fluorescence/confocal microscopy, combined with mass spectrometric methods and mass spectrometric tracking, is increasingly used to assess actual nanoparticle delivery efficiency and refine carrier design for DDRi payloads [294,295].

#### 5.9.3 Safety, Immunogenicity, and Off-Target Effects

Nanocarriers are not intrinsically inert. Chronic accumulation in the liver or spleen can induce inflammation or fibrosis, and surface chemistries such as PEGylation may trigger complement activation-related pseudoallergy (CARPA) or anti-PEG antibody formation, complicating safety profiles [296,297]. Because encapsulation does not eliminate the intrinsic cytotoxicity of DDRis, premature drug release may still damage normal tissues [296]. Preclinical evaluation using 3D microphysiological systems (MPS) and organ-on-chip (Ooc) platforms is therefore critical to define safe pharmacokinetic windows before human translation [253,298].

#### 5.9.4 Regulatory Pathways and Clinical Translation

An overarching goal of research on nanocarrier systems is to improve unified global guidance and to emphasize early characterization of critical material attributes, particle stability, and immunological risk [299]. Additionally, establishing *in vitro*-*in vivo* correlation for release kinetics, defining bioequivalence criteria, and ensuring long-term toxicology data still impose significant challenges [300]. Moreover, the clinical success of approved nanomedicines liposomal doxorubicin, albumin-bound paclitaxel, and liposomal irinotecan, illustrates the relevance of manufacturing control and pharmacovigilance [301–304]. In the future, applying these lessons to DDRi nanocarriers will demand precise control of dual-drug ratios, robust stability under physiological conditions, and transparent reporting of biodistribution and immune responses.

Overall, advances in AI-assisted formulation design, scalable microfluidic synthesis, and improved analytical standards are poised to enhance reproducibility and accelerate regulatory review. It needs to be emphasized that, through early regulatory engagement, standardized safety testing, and comparative evaluation against free drugs, nanocarrier-based DDR inhibitors can evolve from experimental constructs into reproducible, patient-ready therapeutics.

## 6 Emerging Strategies and Future Directions

**Figure 5:**
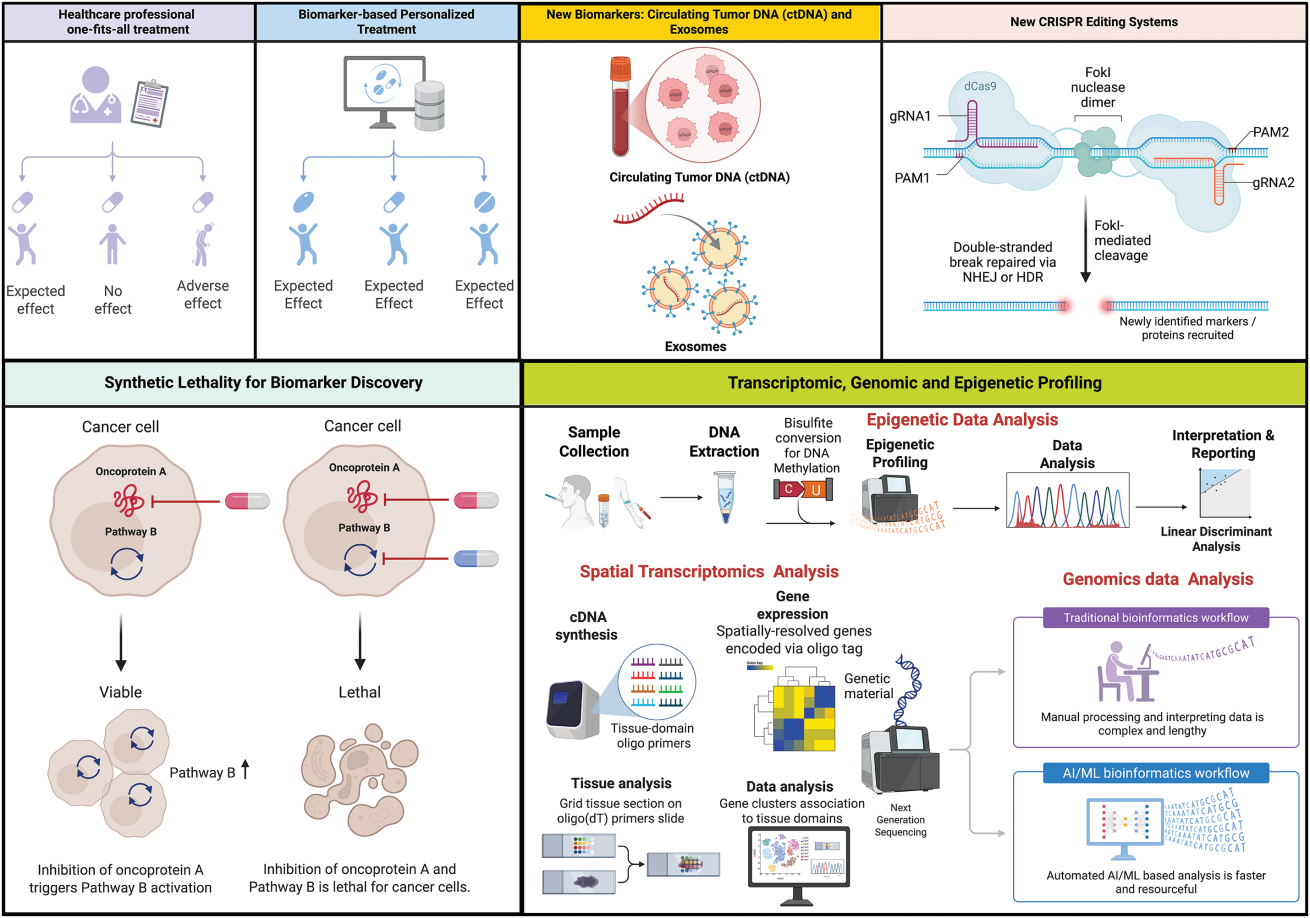
Schematic representation of emerging strategies offering enhanced disease detection and treatment outcomes to overcome long-term treatment resistance

As we elucidate the therapeutic potential of DNA damage inhibitors in the modulation of cancer growth in preclinical and clinical studies, the horizon of DNA damage-targeting therapies continues to expand (Fig. 5). However, realizing the full therapeutic promise of DNA damage-targeting therapies requires a comprehensive and multidimensional future research and experimental models. Additionally, the following areas require focused attention to enhance the translational potential of DDR therapies:

### 6.1 Biomarker-Driven Patient Selection for DDR Inhibitor Therapy

DDR represents an essential cellular network responsible for maintaining genomic stability, whose dysregulation marks cancer presence [305]. Cancer cells often exhibit DDR gene deficiencies alongside replicative stress, making DDR pathways compelling therapeutic targets. Inhibiting DDR processes can induce excessive DNA damage, leading to cell death, particularly in cells with pre-existing DDR defects. PARPis have demonstrated the effectiveness of this approach, especially in tumors with BRCA mutations or homologous recombination deficiencies [305]. However, to expand the successful application of DDRis, refined strategies for patient selection are essential, with a focus on identifying predictive markers that go beyond simple BRCA mutations.

### 6.2 BRCA 1/2 Mutations and Beyond

Mutations in the BRCA1/2 genes are well-established predictors of sensitivity to PARP inhibitors. These mutations impair HR repair, rendering cancer cells more vulnerable to PARPis. However, a significant proportion of tumors without BRCA 1/2 mutations also exhibit DDR deficiencies and may respond to DDRis. Current research focuses on identifying non-BRCA-mutant tumors with potential responsiveness. Future clinical studies could assay key cancer-affected molecules to improve patient stratification for targeted therapy benefits [306].

### 6.3 Genomic Signatures: HRD Scores, RAD51 Foci and Related Markers

Genomic signatures, such as HR deficiency (HRD) scores, can refine patient selection for DDR inhibitor therapy. HRD scores assess the overall level of genomic instability resulting from defects in HR. These scores can identify tumors with BRCAness, a state of HRD that mimics the effects of BRCA 1/2 mutations [307]. Additionally, sequencing chromatin signatures of RAD51 and other assays, such as foci formation and immunoprecipitation, can be used to investigate RAD51-associated factors and assess the functional status of homology-directed repair (HDR). Tumors with impaired RAD51 foci formation are more likely to be sensitive to DDRis. Moreover, Genetic PD-L1 has also been recently shown to regulate HR-mediated end resection, where it regulates BRCAness and impacts synthetic lethality with PARPis, albeit some MAPK and mTORC1 signals are promoted PD-L1 surface receptors [308]. This indicates a new target for anticancer therapies: synthetic lethality approaches that mitigate alternative signaling pathways promoting metastasis.

### 6.4 Refined In Vitro Tools to Study New Biomarkers

Currently, *in vitro* assays utilizing Tus/Ter replication fork barriers to study short tract gene conversions (STGC) and long tract gene conversions (LTGC) in HDR [309], and I-SceI endonuclease reporters to study DSBs [310], provide invaluable information to identify several other regulatory markers other than repair factors that may recruit and stabilize DNA repair in cancer cells, thus defining futuristic models to treat severe malignancies and develop new clinical strategies. New CRISPR-based editing systems and enzymes are an interesting approach for identifying and mitigating new targets in cancer therapies [311–313].

### 6.5 Circulating Tumor DNA (ctDNA) and Exosome: The Emerging Biomarkers

Circulating tumor DNA (ctDNA) and exosomal markers are promising for real-time monitoring of DDRi efficacy [314,315]. CtDNA, or tumor-derived DNA, circulates in the bloodstream, and its levels can reflect the tumor burden and response to therapy [316]. Changes in ctDNA levels during DDRi treatment can provide early indications of drug efficacy or resistance. Also, exosome markers, such as DDR proteins or mutated DNA, can be used to trace DDRi response. For instance, exosomes have been implicated in mediating HR repair-directed end resection and recruiting RPA and RAD51 [317,318]. Therefore, ctDNA and exosomes represent novel therapeutic targets for enhancing DDRi treatment response.

### 6.6 Increased Drug Efflux

Acquired resistance emerges through enhanced drug efflux pump expression, including ABC transporters, which actively expel DDRis from cells, thus lowering their intracellular levels and reducing therapeutic effectiveness [319]. The ATP-binding cassette (ABC) transporter superfamily comprises membrane proteins that utilize ATP hydrolysis to transport a wide range of substrates across cellular membranes actively. Overexpression of these transporters can significantly decrease the intracellular concentration of DDRis, preventing them from effectively targeting their intended molecular targets. For instance, gram-negative bacteria primarily resist antibiotics through drug efflux systems that work together with their outer membrane’s low permeability barrier and additional mutational and plasmid-borne resistance mechanisms [320]. These pumps exploit proton-motive force depletion across the IM to transport diverse substrates through the outer membrane (OM) while requiring accessory proteins for their operation which constitutes their major mechanistic advantage.

### 6.7 Altered DNA Repair Pathway Choice

Cancer cells can also develop resistance by altering their DNA repair pathway choice, shifting from HR to alternative, less precise pathways, such as NHEJ or polymerase theta-mediated repair [319]. These alternative pathways allow cancer cells to bypass the need for HR, which is the primary target of many DDRis, enabling them to repair DNA damage and survive despite DDR inhibition. Since the loss of the canonical DSB repair function (for example, due to defects in HR or altered checkpoint control, as in TP53-mutant tumors) forces the tumor to rely on alternative end-joining pathways, the principal path is microhomology-mediated end joining (MMEJ), which is driven by DNA polymerase theta (POLθ). This interesting target has been extensively studied more recently for its therapeutic potential [321]. Furthermore, POLθ is commonly overexpressed in HR-deficient and TP53-mutant cancers, where it is mechanistically linked to therapeutic resistance (e.g., escape from PARP inhibitors or genotoxic stress) by providing a mutagenic backup repair pathway that tolerates unresolved DSBs [322]. Therefore, several investigations show promising pharmacologic inhibition of POLθ by several chemotypes, including ART558 and novobiocin-like compounds and newer trapping inhibitors that suppress MMEJ and deplete PARP-inhibitor-resistant tumors from their acquired survival mechanism and are synthetically lethal with HR defects [323,324]. Similarly, combined targeting of POLθ and other DSB-repair nodes, such as DNA-PKi, NHEJ, HR inhibitors, and checkpoint kinase inhibitors, unleashes resection-dependent DNA damage in TP53-deficient backgrounds and provides new therapeutic outcomes by exploiting synthetic lethality against compensatory mechanisms.

### 6.8 Transcriptomic and Epigenetic Profiling

This method permits simultaneous identification of DDR weaknesses and counter-resistance strategies. Gene expression analysis identifies DDR deficiencies while DNA methylation together with histone modifications, represent epigenetic changes that alter chromatin accessibility and affect repair protein recruitment. These adaptations enable resistance through compensation for blocked DDR pathways or by activating alternative survival mechanisms. Through DNMT inhibition, scientists explore a re-sensitization approach, while HDAC inhibition boosts immune responses through enhancer reprogramming [319,325].

### 6.9 Lack of Standard Validation

A significant number of DDR-related biomarkers do not have standardized clinical validation which restricts their regular application. The identification of promising biomarkers demands extensive evaluation through future clinical trials before they can be applied in clinical settings. The process entails developing uniform assays alongside establishing optimal cutoff points while assessing biomarker predictive value across varied patient groups.

### 6.10 Need for Comprehensive Assays

Comprehensive genomic and functional assays are needed to guide therapy selection. Given the complexity of DDR pathways and the diverse mechanisms of DDR inhibitor resistance, single biomarkers maybe insufficient to predict treatment response accurately. Comprehensive assays that integrate genomics, spatial transcriptomics, epigenetics, and functional data are needed to provide a more complete picture of the DDR status of a tumor. These assays can help identify patients who are most likely to benefit from specific DDRis or combination therapies.

### 6.11 Exploiting Synthetic Lethality for Biomarker Discovery

The concept of synthetic lethality, where the synergistic efficacy of two or more defects leads to cell death, is central to the development of DDRis. Identifying new synthetic lethal interactions involving DDR pathways can uncover novel biomarkers for DDR response. For instance, the combination of ATR and PARPis has shown promise in IDH1/2-mutant cancers [326], suggesting that IDH1/2 mutations could serve as biomarkers for this combination therapy. Further, microtubule targeting agents (MTAs) have recently been implicated to target DNA DSB repair machinery and have shown significant efficacy to synergize with chemotherapy drugs and target tumor cells [327–329]. The essential function of microtubules, together with proteins such as γ-tubulin, kinesins, and intermediate filaments like vimentin, in sustaining cell structure and enabling inter-organelle communication has gained new significance through recent findings that connect these cytoskeletal elements to DSB repair, thereby creating novel opportunities in cancer research [328–330]. The role of these structural regulators as modulators of the DNA repair pathway highlights their therapeutic role in enhancing the efficacy of DNA damage treatment.

### 6.12 Cancer-Specific Considerations

In the case of pancreatic cancers, approximately 5%–10% of cases have DDR mutations, such as those in BRCA1 and BRCA2, which can be targeted with PARPis [249]. Defining the BRCAness phenotype in pancreatic cancer may be limited to patients harboring DDR genetic alterations [331]. For instance, olaparib demonstrated restricted antitumor effects in advanced platinum-sensitive pancreatic cancer patients with DDR genetic alterations, indicating potential treatment possibilities for specific patient groups [331]. Also, a considerable number of metastatic prostate cancer patients show germline mutations in DDR genes [332]. In a study, the presence of germline BRCA2 mutations negatively affected mCRPC patient outcomes, which showed dependence on the initial treatment approach used. The process of determining germline BRCA2 status can help select initial treatments for mCRPC [332].

### 6.13 Accelerating DDR-Targeted Precision Oncology

The advent of transformative technologies such as multi-omics, functional genomics, and computational modeling is redefining how the DDR is studied and therapeutically exploited. Representative studies demonstrate that these emerging platforms could reveal cellular heterogeneity, predict drug response, and reproduce patient-specific tumor complexity. Incorporating such technologies into DDR research is essential to overcome translational bottlenecks and to align DDRi development with the broader evolution of precision oncology. The merging role of spatial/single-cell omics, CRISPR screens, AI/ML predictive modeling, organoid/PDX systems, and an integrated translation framework is discussed in the present section.

#### 6.13.1 Spatial and Single-Cell Omics Reveal DDR and TME Heterogeneity

Single-cell and spatial-omics approaches now expose the cellular diversity and microenvironmental context of DDR activation within tumors [333]. Platforms such as 10× Visium, MIBI, and CODEX imaging quantify phosphorylated markers (γH2AX, pCHK1) at subcellular resolution and map their distribution across hypoxic and immune niches [334,335]. There is a growing body of work combining transcriptomic, proteomic, and metabolic imaging to investigate “DDR-addicted” regions [336,337]. For example, combining transcriptomic, proteomic, and metabolic imaging has revealed that replication stress and oxygen gradients coincide with immune-exclusion areas that are most susceptible to DDR inhibition or nanoparticle-based delivery [338]. These data-driven atlases bridge molecular profiling and spatial pathology, allowing patient-specific mapping of DDR dependencies.

#### 6.13.2 CRISPR Functional-Genomics Screens Define Synthetic-Lethal Networks

Genome-scale CRISPR/Cas9 and CRISPRi/a screens systematically identify vulnerabilities that condition DDRi sensitivity and resistance [339]. Recent studies have pinpointed POLQ as a synthetic-lethal partner of DNA-PK inhibition, MYT1 up-regulation as a cause of WEE1i resistance, and CDK8/Cyclin C loss as a determinant of ATRi escape [147,340,341]. Such discoveries clarify compensatory repair pathways and enable rational drug-combination design. When coupled with base-editing or enhancer-targeting platforms, CRISPR screens also map non-coding and regulatory elements that govern DDR gene expression, thereby enriching biomarker discovery for clinical translation [342].

#### 6.13.3 Artificial Intelligence and Machine Learning for Predictive Modeling

As knowledge advances and artificial intelligence- and machine-learning-based mathematical models improve, such studies may significantly increase our understanding of DDRi response. Artificial-intelligence frameworks now integrate vast multi-omic and pharmacogenomic datasets to predict DDRi response [343,344]. For instance, models trained on DepMap and GDSC2 were found to correlate genomic instability signatures, homologous recombination deficiency scores, and transcriptomic features with therapeutic sensitivity and toxicity [345]. In drug design, deep-learning algorithms have also been reported to accelerate the optimization of kinase inhibitors, thereby enhancing selectivity and blood–brain barrier penetration [346,347]. At the same time, *in-silico* “digital-twin” simulations are rapidly advancing in tumor therapy via optimizing personalized treatment [348]. In the future, digital twin technology combined with genomic, radiomic, and pharmacokinetic derived inputs could ensure safety of DDRi regimens virtually before clinical exposure. Overall, these computational advances might improve and convert descriptive biology into predictive precision.

#### 6.13.4 Patient-Derived Organoids and Xenografts Enable Functional Precision Testing

Patient-derived organoids (PDOs) and xenografts (PDXs) faithfully preserve tumor heterogeneity and microenvironmental cues, providing translational platforms to validate DDRi activity [349,350]. In the past few years, PDO biobanks from breast, ovarian, colorectal, and pancreatic cancers have demonstrated that replication stress levels and stromal interactions shape drug response [351]. Additionally, co-culture experiments with fibroblasts or immune cells have proven beneficial for recapitulating resistance mechanisms driven by cytokine or extracellular matrix signaling [352]. In the future, combining CRISPR perturbation with high-content imaging would enable researchers to assess synergy and toxicity in real time, effectively linking genomic prediction to pharmacologic validation.

In summary, an integrated use of spatial mapping, CRISPR functional genomics, AI analytics, and patient-derived modeling might allow a mechanistic resolution, predictive biomarkers, and experimentally testable therapeutic hypotheses. In the future, as integration expands to incorporate circulating tumor DNA dynamics and radiomic imaging, DDR-targeted therapy will transition from empirical evaluation to a data-driven paradigm in which each patient’s genomic instability landscape and microenvironmental architecture guide optimal inhibitor selection and combination strategies.

### 6.14 Effect of Gut Microbiome and Epigenetic Modulators on the Therapeutic Efficacy of DDR Inhibitor Response

In recent years, studies have reported that tumor-intrinsic alterations alone cannot fully explain the heterogeneity in therapeutic responses to DDRis. For example, the gut microbiome and the epigenetic landscape profoundly impact key signaling pathways related to the host immune system, metabolism, as well as chromatin architecture, thereby modulating DNA repair capacity, drug pharmacokinetics, and overall treatment efficacy. A better understanding of altered molecular networks is crucial for refining biomarker discovery and developing integrative precision oncology strategies.

#### 6.14.1 Gut Microbiome as a Determinant of DDRi Efficacy and Toxicity

The intestinal microbiome regulates systemic inflammation, oxidative stress, and immunogenic signaling, all of which intersect with DDR and antitumor immunity [353]. Metabolites such as short-chain fatty acids (butyrate, propionate) and tryptophan-derived indoles regulate histone acetylation and type I interferon pathways, indirectly amplifying the cGAS–STING cascade triggered by DDR inhibition of commensal species, including *Akkermansia muciniphila* and *Bifidobacterium* spp [354]. An elevated immune activation has been associated with enhanced responses to PARP and ATR inhibitors [355,356]. At the same time, antibiotic-induced dysbiosis has been reported to dampen cytotoxic T-cell infiltration and increase gastrointestinal toxicity [357]. Additionally, microbial enzymes also modify NAD^+^ metabolism and drug conjugation, influencing the activity of NAD-dependent enzymes such as PARP [358]. These findings indicate that modulation of gut microbiota via dietary intervention, probiotics, and fecal transplantation could reduce systemic inflammation and enhance DDRi efficacy.

#### 6.14.2 Epigenetic Regulation of DNA Repair and DDRi Sensitivity

Epigenetic control of chromatin structure determines accessibility of repair proteins and directly affects DDR pathway selection. Several studies show that promoter hypermethylation regulates genes involved in DNA repair pathways, such as BRCA1, MLH1, and RAD51C, potentially inducing a “BRCAness” phenotype that makes them sensitive to specific treatments, such as PARPi and ATRi, and enhances their sensitivity to these agents [359]. On the contrary, global hypomethylation promotes error-prone end joining and drug resistance. DNA-methyltransferase inhibitors (DNMTis) can reverse gene silencing and induce viral-mimicry responses by reactivating endogenous retroelements, thereby enhancing type I interferon signaling and immunogenic cell death when paired with DDR blockade [360,361]. Similarly, HDACis impair homologous recombination by disrupting RAD51 and 53BP1 assembly, thereby sensitizing tumors to PARP, WEE1, and ATR inhibitors [362,363]. Conversely, repressive marks such as H3K9me3 stabilize heterochromatin and confer radio resistance [364]. Non-coding RNAs, including *LINC00460* and *miR-182*, add another layer of control by modulating molecular circuits related to immune response, checkpoint activity, and therapy response, thereby influencing tumor progression and synthetic-lethal responses [365,366].

#### 6.14.3 The Interplay of Microbial and Epigenetic Modulation in Precision DDR Therapy

The interaction between microbial metabolites, immune tone, and chromatin remodeling establishes an epigenetic–metabolic axis that determines DDRi response [367]. Integrative profiling of fecal metabolites, circulating short chain fatty acids (SCFAs), and chromatin marks, such as LINE-1 methylation and histone H3 acetylation, may yield composite biomarkers of sensitivity and resistance [368]. Ongoing trials exploring combinations of DNMTis or HDACis with PARPi or ATRi aim to resensitize tumors that have become resistant [369]. At the same time, microbiome-directed interventions seek to maintain immune responsiveness during chronic DDR-targeted therapy [370,371]. Emerging areas of metagenomic sequencing, single-cell epigenomics, and AI-based modeling might pave the way for personalized DDRi regimens. Utilizing them in combination might fill a significant gap by addressing questions specific to each patient’s microbial and epigenetic landscape, providing a framework that links systemic biology with data-driven precision oncology.

## 7 Conclusion

Based on our insights, the DDR pathways represent a critical axis in maintaining genomic integrity and an increasingly valuable target in cancer therapy, with a focus on developing advanced inhibitors. Established strategies, particularly the use of PARPis in HR-deficient tumors, have demonstrated the therapeutic potential of exploiting synthetic lethality. Similarly, SMIs of key DDR kinases, such as ATM, ATR, and DNA-PK, have expanded the therapeutic landscape, although clinical efficacy remains hindered by both intrinsic and acquired resistance mechanisms. Therapeutic advancements in DDR-targeting now feature advanced and site-specific inhibitors designed to surpass existing treatment barriers while broadening synthetic lethality uses. The development of NP-based delivery systems is boosting DDRis’ precision and bioavailability while facing persistent toxicity challenges and concerns.

The response to DDRi treatments depends not only on tumor-intrinsic vulnerabilities but also on the complex interactions within the TME and immune landscape. A combination of immune infiltration, hypoxia, and stromal interactions drives resistance, which demands a transition to therapies that blend immunomodulatory agents with DDRis. The identification of promising biomarkers demands extensive evaluation through future clinical trials before they can be applied in medical settings. The process entails developing uniform assays alongside establishing optimal cutoff points while assessing biomarker predictive value across varied patient groups.

Future research should focus on understanding resistance through rational drug combinations, improving delivery platforms, and harnessing DDRis to enhance antitumor immunity using future technologies like AI-driven platforms. Advancing the clinical utility of DDR-targeting agents will require not only a deeper understanding of DDR biology but also integrated efforts to characterize the influence of the TME and immune system. Continued exploration of synthetic lethal interactions, biomarker discovery, and clinical trials involving combination regimens will be key to translating DDR inhibition into durable and widespread cancer therapies.

## Data Availability

Not applicable.

## Abbreviations

ATM: Ataxia-telangiectasia mutated
ATMi: ATM inhibitor
ATRi: ATR inhibitor
BER: Base excision repair
CHK: checkpoint kinases
DOX: Doxorubicin
DDAs: DNA-damaging agents
DDR: DNA damage response
DDRi: DNA damage response inhibitors
DNA-PK: DNA-dependent protein kinase
DNA-PKis: DNA-PK inhibitors
EGFR: Epidermal growth factor receptor
HDACis: Histone deacetylase inhibitors
HR: Homologous recombination
HIFs: Hypoxia-inducible factors
IDL: Insertion/deletion loop
IFN: Interferon
Ku-DBis: Ku-DNA binding inhibitors
NER: Nucleotide excision repair
NHEJ: Non-homologous end joining
PD-L1: Programmed death-ligand 1
PIKK: Phosphatidylinositol 3-kinase-related kinase
Tregs: Regulatory T cells
SMIs: Small-molecule inhibitors
SCLC: Small cell lung cancer
SSBs: Single strand breaks
ssDNA: Stranded DNA
TGF-β: Transforming growth factor beta
TLS: Translesion synthesis
WEE1: WEE1 G2 checkpoint kinase
